# Treatment adherence in acne vulgaris: systematic review and narrative synthesis

**DOI:** 10.1093/skinhd/vzag084

**Published:** 2026-07-31

**Authors:** Miriam Santer, Emma Maund, Taeko Becque, Rosie Essery, John S Barbieri, Beth Stuart, Tracey H Sach, Fathema Miah, Matthew J Ridd, William Price, Joanna Pang, Sophie Dove, Irene Soulsby, Kim S Thomas, Kate Henaghan-Sykes, Mary Steele, Steph Easton, Sebastien Pollet, Angel Umensofor, Sinéad M Langan, Nick A Francis, Andrew R Thompson, Paul Little, Alison M Layton, Ingrid Muller

**Affiliations:** Primary Care Research Centre, University of Southampton, Southampton, UK; Primary Care Research Centre, University of Southampton, Southampton, UK; Primary Care Research Centre, University of Southampton, Southampton, UK; Primary Care Research Centre, University of Southampton, Southampton, UK; Advanced Acne Therapeutics Clinic, Brigham and Women’s Hospital, Harvard Medical School, Boston, MA, USA; Pragmatic Trials Unit, Queen Mary University of London, London, UK; Primary Care Research Centre, University of Southampton, Southampton, UK; Primary Care Research Centre, University of Southampton, Southampton, UK; Centre for Applied Excellence in Skin & Allergy Research, University of Bristol, Bristol, UK; Pharmacy Department, East Lancashire Hospitals NHS Trust, Burnley, UK; Primary Care Research Centre, University of Southampton, Southampton, UK; Public Contributor, University of Southampton, Southampton, UK; Public Contributor, University of Southampton, Southampton, UK; School of Medicine, University of Nottingham, Nottingham, UK; Primary Care Research Centre, University of Southampton, Southampton, UK; Primary Care Research Centre, University of Southampton, Southampton, UK; Primary Care Research Centre, University of Southampton, Southampton, UK; Primary Care Research Centre, University of Southampton, Southampton, UK; School of Medicine, University of Nottingham, Nottingham, UK; Faculty of Epidemiology and Population Health, London School of Hygiene and Tropical Medicine, London, UK; Primary Care Research Centre, University of Southampton, Southampton, UK; Clinical Psychology, Cardiff and Vale University Health Board, Cardiff, Wales, UK; Clinical Psychology, Cardiff University, Cardiff, UK; Primary Care Research Centre, University of Southampton, Southampton, UK; Dermatology, Harrogate and District NHS Foundation Trust, Harrogate, UK; Dermatology, Hull York Medical School, University of York, Hull, UK; Primary Care Research Centre, University of Southampton, Southampton, UK

## Abstract

**Background:**

Acne vulgaris is common and has a substantial impact on patients’ quality of life. Low adherence to acne treatments is a barrier to successful management.

**Objectives:**

To examine the prevalence of adherence to oral and topical treatments for acne, factors associated with adherence and reasons for nonadherence.

**Methods:**

We included primary quantitative studies that investigated acne-related experiences and behaviours, including predictors, determinants and outcomes associated with treatment adherence. We searched the following databases from inception until 24 March 2025, with no language restrictions: MEDLINE, PubMed, Embase, PsycINFO and CINAHL. Quality was appraised using the Mixed Methods Appraisal Tool. We present the findings related to observational studies, analysed using narrative synthesis.

**Results:**

Twenty-six eligible papers reported studies in the Americas, Europe and Asia. Thirteen papers reported quantitative descriptive studies (8111 survey participants plus 2 studies that reported data from electronic health records) and 13 papers (12 studies, 37 884 participants) reported other designs such as analytic cross-sectional or cohort studies. Methods for evaluating adherence varied: 21 studies used self-report with differing questionnaire designs, 3 used prescription data to evaluate medication possession ratio, 3 assessed drug refills, 1 carried out database drug survival analysis and 1 used pill count or tube weighing. Four studies used more than one method. The prevalence of adherence estimates varied from 6% to 84%, with larger studies estimating 50–65% adherence. Adherence was lower among younger people, but there was inconsistent evidence for differences between men and women, or between topical and oral treatments. Reasons for nonadherence included cost, side effects, perceived treatment ineffectiveness or symptom resolution.

**Conclusions:**

Improving treatment adherence in acne remains a priority, particularly for younger people. Interventions addressing the management of side effects and time to effectiveness of treatments may be beneficial.

## Teaser

This systematic review examines prevalence of treatment adherence for oral and topical treatments for acne, factors associated with treatment adherence and reasons given for nonadherence. Twenty-six eligible papers were included, with estimates of treatment adherence prevalence varying from 6% to 84%, although larger studies estimated 50–65%. There was lower adherence amongst young people aged less than 15 years but little evidence for differences in adherence between men and women, or between topical and most oral treatments. Common reasons for nonadherence included cost, side effects, perceived ineffectiveness of treatments or resolution of acne symptoms.

What is already known about this topic?

- Acne is common and has a substantial impact on patients’ quality of life.
- Low treatment adherence is a major barrier to effective treatment of acne.

What does this study add?

- Prevalence estimates for acne treatment adherence vary widely, from 6% to 84%, with many different methods of assessment.
- Younger people (aged <15 years) appear to have lower adherence to treatment, but there is little consistent evidence for differential adherence between men and women, or between topical and most oral treatments.
- Interventions to improve treatment adherence should address common concerns around side effects and perceived ineffectiveness.

Acne vulgaris (or acne) is common, affecting 90% of teenagers at some point.^1^ In approximately 50% of people, acne persists for several years and in approximately 20% it leads to scarring.^2,3^ Acne can have a substantial impact on patients’ quality of life, including an increased risk of depression and suicidal thoughts.^2^ Antimicrobial resistance, resulting from long courses of oral antibiotics for acne, is a concern.^4^

There is good evidence for the effectiveness of topical treatments for acne, such as topical retinoids, benzoyl peroxide (BPO) or topical antibiotics (used alongside topical retinoid or BPO), and these are widely recommended as the first-line treatment for mild-to-moderate acne.^5,6^ However, for medication to work it must be taken or used regularly for several weeks.^7^ Nonadherence is a hidden problem in many conditions, with treatment underuse being a barrier to improved outcomes.^8^ Treatment nonadherence has been categorized into issues around ‘initiation’, ‘implementation’ and ‘discontinuation’.^9^ There is a common perception that there is a particular problem with low treatment adherence to topical treatments for acne,^2^ but, to date, there has been relatively little exploration of whether the main barriers to treatment adherence in patients with acne are around ‘initiation’, ‘implementation’ or ‘discontinuation’.

A 2014 systematic review on adherence to acne medications in randomized controlled trials (RCTs) found adherence rates ranging from 7% to 98%, but the authors commented on methodological weaknesses and the fact that estimates from trial participants are likely to differ from other populations.^10^ A recent narrative review of methods used to measure adherence to dermatological treatments in general found financial considerations to be the most frequently mentioned barrier.^11^ This review also concluded that, although topical treatments are generally thought to be associated with lower adherence across a range of conditions, the evidence for this is weak in acne.^11^

We initially aimed to carry out a systematic review to review and synthesize the quantitative literature investigating people’s acne-related experiences and behaviours, including treatment adherence, help-seeking and personal expenditure. However, the breadth of the evidence was such that this paper focuses on observational studies that report adherence to topical and oral treatments for acne, addressing the following questions:

- How is treatment adherence in acne measured?
- How good is treatment adherence to oral and topical treatments for acne?
- What is known regarding the factors associated with treatment adherence for acne?
- What reasons are commonly given for nonadherence to acne treatments?

## Materials and methods

### Protocol and registration

The review protocol was registered with the PROSPERO International Prospective Register of Systematic reviews (https://www.crd.york.ac.uk/PROSPERO/view/CRD42022377900).

### Search strategy and information sources

We searched the following databases from inception until 2 June 2023: MEDLINE (OvidSP), PubMed, Embase (OvidSP), PsycINFO (EBSCOhost) and CINAHL (EBSCOhost). The same searches were rerun on 24 March 2025 to update the review.

The MEDLINE search strategy (Appendix S1; see Supporting Information) was developed first and then reviewed by an experienced health librarian. This strategy was then adapted by one researcher for the remaining databases (see Appendix S1). Experts in the field were consulted to ask whether they were aware of any additional relevant studies.

### Study selection

Two reviewers independently screened all titles and abstracts using the eligibility criteria stated below, with disagreements resolved through discussion and arbitration with a third reviewer if necessary. We obtained full articles where titles or abstracts met the inclusion criteria. One researcher independently assessed whether these full papers met the inclusion criteria, with a 40% sample of full-text papers screened by a second reviewer, as well as whenever the first researcher was uncertain. Any disagreements were resolved by discussion and arbitration with a third reviewer. See Appendix S2 (Supporting Information) for a list of studies excluded on the basis of full-text screening, along with reasons for exclusion.

### Eligibility criteria

The planned systematic review included primary studies that used quantitative data collection or analysis methods and presenting quantitative data (descriptive and/or inferential statistics), either standalone or as a distinct part of a mixed-methods study investigating acne-related experiences and behaviours, including treatment adherence, help-seeking and personal expenditure. However, due to the large number of studies retrieved, and their clinical and methodological heterogeneity, this paper reports on primary studies that reported data on treatment adherence. Furthermore, given the previous systematic review of clinical trials,^10^ and likelihood of trial participants being less reflective of ‘real-life’ adherence, this paper focuses on ­non-RCTs only. Examples of nonrandomized studies include ­nonrandomized experimental studies, analytical cross-sectional ­studies, case–control studies, cohort studies, or before-and-after studies [following the Mixed Methods Appraisal Tool (MMAT) algorithm for selecting study categories].^12^ A systematic review of RCTs of interventions to promote treatment adherence will be published separately. For information purposes, the retrieved RCTs are presented in Appendix S3 (see Supporting Information), and papers included for the systematic review of RCT evidence are listed in Appendix S4 (see Supporting Information).

#### Population

We included studies of people of all ages with acne, provided at least some participants were aged between 13 and 25 years (their data did not need to be reported separately), health practitioners treating acne and carers or parents of children with acne. We included observational studies concerning multiple skin conditions if data for acne alone were reported separately. We excluded general public samples where it was unclear what proportion of the sample had acne and where their data were not reported separately.

#### Exposures

In the original planned systematic review, papers eligible for inclusion were study designs exploring (i) acne-related beliefs and behaviours:

- Acne-related beliefs, perceptions or preferences, and adherence to acne treatments
- Acne-related beliefs, perceptions or preferences, and acne help-seeking behaviour
- Acne-related beliefs, perceptions or preferences, and health professionals’ acne-related treatment or management decisions, or behaviour

and (ii) acne-related behaviours and outcomes:

- Treatment adherence and acne-related outcomes
- Help-seeking behaviour and acne-related outcomes
- Healthcare professionals’ acne-related treatment management decisions and behaviour, and acne-related outcomes.

We excluded studies concerning only oral isotretinoin as a previous systematic review has shown good treatment adherence to oral isotretinoin,^10^ although we included studies where isotretinoin was one of several treatments. We excluded studies concerning the mental health impact of acne alone, unless this was related to treatment adherence. We excluded studies concerning acne scarring or postinflammatory hyperpigmentation as the treatments and related adherence issues differ substantially from the treatment of active acne. As described above, given the volume and heterogeneity of studies identified, this paper reports on studies that included data on adherence to topical and oral treatments for acne.

#### Outcomes

In the initial planned systematic review, papers of interest reported quantitative data relating to predictors of, correlates of or factors associated with any of the following outcomes:

- Acne symptom severity
- Acne-related quality of life as measured by patient-reported outcome measures, e.g. ACNE-QoL, CompAQ, ACNE-Q, etc.
- Adherence/compliance to or engagement with acne treatments or interventions.
- Acne help-seeking behaviour in relation to acne treatment and management
- Knowledge and/or beliefs about acne
- Healthcare professionals’ acne treatment or management-­related decisions or behaviours
- Out-of-pocket costs incurred by young people and their families
- And/or relationships between adherence and/or engagement with the intervention, and any of the other outcomes specified.

The results from our systematic searches regarding published papers on personal expenditure, beliefs and help-seeking are included in Appendix S5 (see Supporting Information).

#### Language and publication types

There were no language restrictions. Articles written in a language other than English were translated into English. We excluded studies reported in conference abstracts only as scoping revealed that these were unlikely to contain the quantitative outcome-related data of interest. We excluded case studies.

#### Data extraction process

Data extraction was performed by one researcher into a standardized prepiloted form created in Microsoft Excel. Extracted data were checked by a second researcher.

#### Assessment of study quality

Each study was assessed by one researcher against the MMAT.^12^ All assessments were then checked by a second researcher. Any disagreements were resolved through discussion. No studies were excluded on the basis of quality criteria, but assessments were used to guide confidence in study findings.

#### Data synthesis

This paper reports a narrative synthesis^13^ focused around addressing the following more specific research questions:

- How is treatment adherence in acne measured?
- How good is treatment adherence for oral and topical treatments for acne?
- What is known regarding the factors associated with treatment adherence for acne?
- What reasons are commonly given for nonadherence to acne treatments?

Precision estimates for prevalence statistics were not attempted, as many studies lacked the necessary detail to facilitate this approach. Due to the different methods of the assessment of treatment adherence, no overall pooled estimate was made. We did not attempt to obtain data from investigators, as many papers included were over 10 years old.

## Results

We identified 5682 individual records for screening and reviewed 359 full-text papers. Of these, 26 papers reporting 25 non-RCT quantitative studies met the inclusion criteria for studies of adherence to acne treatments (Figure 1). (Two papers reported analyses carried out on the same dataset.^15,16^) Papers were from the Americas, Europe and Asia (see Table 1). Following MMAT categorization of study categories,^12^ 13 papers reported quantitative descriptive studies (including a total of 8111 survey participants),^18,21,22,25,28–34,36,38^ 1 reported a cross-sectional study using a database of electronic health records,^18^ and 1 used both electronic health record data and survey data.^39^ Thirteen papers (12 studies) reported designs such as cross-sectional analytic or a study of participants within a cohort study or nonrandomized trials, with a total of 37 884 participants.^15–17,19,20,23,24,26,27,35,37,39,40^

**Figure 1:**
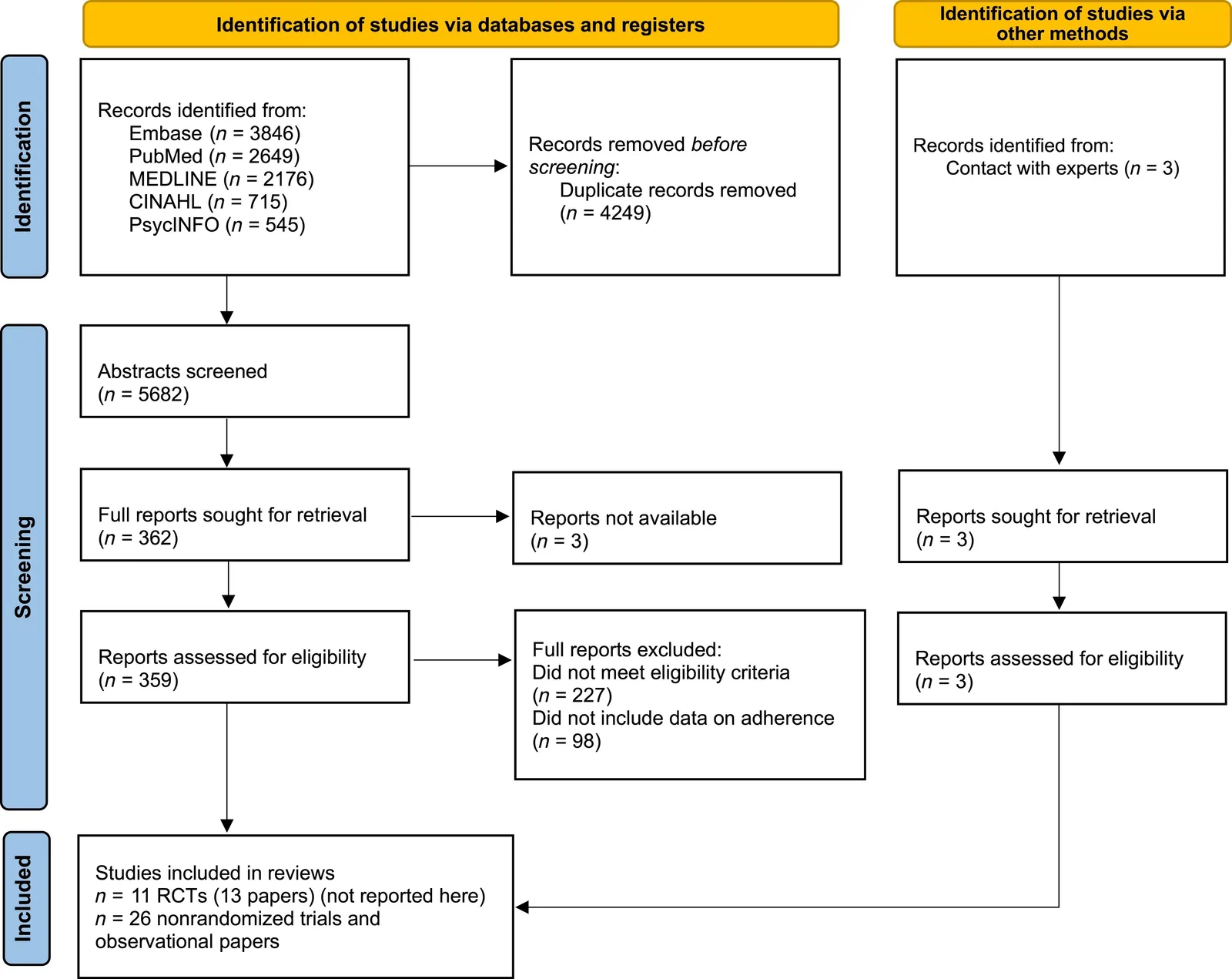
PRISMA 2020 flow diagram. From Page *et al*.^14^ RCT, randomized controlled trial.

**Table 1:** Details of included studies

First author, year	Aim	Design	Country	Source of recruitment	Number of participants, severity (if stated), gender, age, ethnicity (if stated)	Funding or competing interests
Anderson 2015^17^	To determine the frequency of primary nonadherence to acne treatments and to assess the effect of the number of treatments on adherence	Self-report; analytical cross-sectional telephone survey	USA, single centre	Acne diagnosed by a dermatologist in past 3 months	143 patients (47 on 1 medication, 48 on 2, 48 on 3); 103 female, 40 male; mean age 28 years (SD 14)	Unrestricted educational grant from Galderma
Balkrishnan 2006^18^	To investigate the relationship between health status and costs linked with acne treatment, and other aspects related to medication use	Cross-sectional database study (population survey)	USA	People with acne identified with diagnostic code from US Medical Expenditure Panel Survey dataset	Approximately 5.68 million people with acne; 60% female 40% male; 49.1% aged ≤25 years; ethnicity 90.5% White	Supported by a grant from Galderma
Cook-Bolden 2006^19^	To measure effectiveness, satisfaction and preferences around adapalene gel 0.1% but also reports adherence data	Self-report; study within a nonrandomized trial	USA	Participants in the Measuring Acne Outcomes in a Real-World Experience (MORE) trial	443 patients with acne aged ≥12 years; no baseline characteristics	Author is an advisory board member and speaker for Galderma
de Lucas 2015^20^	To evaluate the impact of treatment adherence on clinical outcome, and how the use of adjuvant treatments (e.g. cleansers, moisturizers) influence treatment adherence and acne severity	Self-report; observational noninterventional prospective cohort	Spain, multicentre	Patients recruited by Spanish dermatologists taking part in the ACTUO trial	643 people with mild-to-moderate acne, 62.2% female, 37.8% male; mean age 21.3 years (SD 7.2)	Competing interests: 2 co-authors are ISDIN employees; the ACTUO study was supported by an unrestricted grant of ISDIN SA
Dréno 2010^21^	To assess risk of poor adherence in patients with acne and examine patient and treatment characteristics correlated with adherence	Self-report; cross-sectional survey	3 geographic regions: the Americas (*n* = 952), Europe (*n* = 1196) and Asia (*n* = 1191)	People attending dermatologists for follow-up of acne. Dermatologists selected by random in each participating country from list provided by Galderma. Minimum 105 dermatologists in each country were instructed to enrol minimum 4 patients with acne	3339 participants attending follow-up appointments for acne, gender not stated, mean age 19.6 years (SD 5.2) for male participants and 22.7 years (SD 7.2) for female participants	Data collection was supported by Galderma International
Feldman 2011^22^	To assess the frequency and impact of self-reported dryness and irritation due to clindamycin–BPO (5%) fixed combination acne treatment and how patients with acne cope with dryness and irritation to better understand the implications for adherence and clinical practice	Self-report; cross-sectional questionnaire (online)	USA	People invited from database of individuals who had opted to participate in consumer research after using commercially available treatment. Quotas on age or gender were set to reach a representative sample	200 participants; 57% reported having moderate acne and 28% severe acne; 91 male participants; 109 female participants; age range 15–40 years (100 aged 15–21 and 100 aged 22–40 years); 69.5% White, 13% Hispanic, 8% Black, 8% Asian and 1.5% other	Coria Laboratories, subsidiary of Valeant Pharmaceuticals, funded Inergy’s activities pertaining to this manuscript
Gollnick 2015 ^23^	To assess the efficacy of adapalene–BPO and patient adherence under daily clinical practice conditions	Self-report; nonrandomized observational study (before and after study)	Germany	Participants selected from dermatology practices when adapalene–BPO was prescribed	3171 enrolled (2780 completed the study). Patients with moderate acne; 1420 (51.1%) male, 1326 (47.7%) female, 34 (1.2%) unknown; mean (SD) age 16.3 (2.5) years	Authors linked to, or employed by, Galderma
Gollnick 2015^24^	To evaluate the effect of acne treatment on QoL and treatment adherence under long-term conditions in daily practice	Self-report; noninterventional observational cohort study	Germany	Participants selected from dermatology practices when adapalene–BPO was prescribed	6036 (5131 included) patients with moderate-to-severe facial acne; 56.5% male, 42.8% female, 0.7% unknown; median age 18.0 years	Authors linked to, or employed by, Galderma
Hayran 2021^25^	To investigate treatment adherence and satisfaction of patients with acne and identify independent factors that affect them	Self-report; cross-sectional survey (unclear whether face-to-face but likely so if IGA completed)	Turkey	Patients attending dermatology clinic	500 patients with acne; IGA severity 10.8% almost clear, 30.4% mild, 32.4% moderate, 19.4% severe, 6.6% very severe; 77.4% of the patients were female [female (*n* = 387), male (*n* = 113)], mean (SD) age 21.48 (4.28) years	NR
Hester 2016^16^	To determine whether there are differences in medication adherence rates between children with acne aged 6–11 years and those aged 12–17 years	Analytical; cross-sectional MPR	USA	Assessed administrative claims for Medicaid enrolees from eight US states (1 July 2004–1 July 2007), with at least one claim for an acne-related medication during the study period	2860 children aged 6–11 years; 26.9% male, 73.1% female; 33.4% White, 57% Black, 2.5% Hispanic, 7% other. 7179 adolescents aged 12–17 years; 50.3% female, 49.7% male); 42.4% White, 48.7% Black, 2.7% Hispanic, 6.1% other	NR
Jones-Caballero 2008^26^	To analyse the relationship between self-reported adherence to treatment and QoL	Self-report; questionnaire at baseline and 12 weeks later (before and after study)	Spain	Patients cared for by clinicians in Spain	Data reported on 1871 people with mild or moderate acne; 36.3% male and 63.7% female; mean age 20.13 years; 79.8% White, 19.0% Hispanic, 0.2% gypsy, 0.2% Black, 0.7% others	Supported by Yamanouchi Pharma (Astellas)
Hoai 2021^27^	To investigate the drug survival of topical treatments administered to patients with moderate acne	Nonrandomized drug survival analysis (review of medical records)	Belgium	Patients who received treatment from one university hospital and three private practices in Brussels, who began treatment between January 2010 and December 2019	1160 treatment series in 1029 patients with moderate acne, 36% male; mean age 22.8 years (SEM 0.3)	None
Liu 2022^28^	To explore college students’ perceptions regarding acne and its topical medications, and to evaluate whether these perceptions affect their motivation to seek and adhere to treatments	Self-report; cross-sectional survey (online)	China	Students from one university, with or without self-diagnosed acne	1415 responses, of whom 483 had self-reported acne: 70.0% mild, 24.2% moderate, 5.8%; female 52.2%, male 47.8%; no age data reported other than range 18–24 years	Natural Science Foundation of Jiangsu Province
Miyachi 2011^29^	To determine the likelihood of treatment adherence based on type of treatment (topical or oral) and factors associated with medication-taking	Self-report; cross-sectional survey (self-completed and dermatologist-completed) questionnaires	Japan	Dermatology clinics were members of a panel established by the healthcare marketing research agency conducting the study. Participating dermatologists recruited patients using acne treatment who had a medical consultation for acne ≥1 month to ≤3 months	59 dermatologists recruited 428 patients with acne (20.3% mild, 55.1% moderate, 20.8% severe (Japanese Dermatological Association criteria); 64% female, 36% male; mean age 24.4 years (no SD)	Supported by Galderma
Rapp 2004^30^	To examine the association between trait anger and acne severity, patient satisfaction, adherence to treatment advice and QoL	Self-report; survey (paper or online)	USA	One urban high school and two universities (one public, one private). Respondents were recruited in an academic medical centre dermatology clinic and through a database of people with acne maintained by Johnson&Johnson for the purpose of marketing research	479 participants with facial acne; mean age 23 years (range 16–62); 64% female; 77% White	The study was funded by a grant from Johnson&Johnson
Rendon 2015^31^	To (i) describe and analyse acne treatment patterns and expectations in a cohort of adult female participants of different ethnic backgrounds; and (ii) explore their potential effects on treatment adherence and satisfaction	Self-report; survey (online)	USA	Women with self-reported acne recruited through YouGov Panel Using preregistered US panellists	312 participants with self-reported acne. 36.5% reported none/some redness from facial acne in past 4 weeks, 63.5% had moderate/a lot/extensive; all female; average age was 35.3 years (SD 5.9); 34.3% White, 30.8% Black, 17.6% Hispanic, 17.3% Asian/other. Across the whole sample, 73.4% had prescription drug coverage	Authors declare financial involvement, including consultancies and employment, with a number of pharmaceutical and cosmetic companies who produce products for acne
Salamzadeh 2020^32^	To determine the rate of treatment adherence among patients with acne vulgaris and identify factors affecting it	Self-report; cross-sectional survey	Iran	Patients diagnosed with acne at two dermatology clinics of educational hospitals in Tehran, mostly attending for follow-up	200 participants with self-reported acne severity 11% mild, 41.5% moderate, 47.5% severe; 68% female, 32% male; mean (SD) age 22.13 (5.51) years; 11% without insurance	The authors declare no conflicts of interest
Sevimli Dikicier 2019^33^	To evaluate the choices in topical treatment for acne vulgaris and patient adherence to the prescribed treatment	Self-report; cross-sectional survey (dermatologist-directed questionnaire)	Turkey	Patients with acne who were prescribed topical treatment in past 6 months from one dermatology outpatient clinic in a teaching hospital	250 participants, severity at first visit, 41.6% mild, 54.8% moderate, 3.6% severe (GAGS); female 71.2%, male 28.8%; mean (SD) age 18.6 (2.8) years	Declared no conflicts of interest
Tan 2009^34^	To evaluate the association between sociodemographic factors, clinical severity, QoL impact and adherence to topical acne treatments	Self-report; questionnaire survey	Canada	Participants were people with acne referred from community to dermatologists, were prescribed topical treatment only at first appointment and who returned for follow-up after 2 months	152 participants with facial acne (6.6% clear, 27% almost clear, 48% mild, 15.8% moderate, 2.6% severe), 67% female, mean (SD) age 22.4 (9.4) years, 87% White	This study was supported by unrestricted educational grants from Hoffman-LaRoche Ltd, Berlex, Stiefel and Dermik Laboratories. Drs Lynde and Tan have served as investigators, consultants or speakers for Hoffman La-Roche, Dermik, Stiefel, Berlex and Galderma
Tan 2013^15^	To examine medication adherence, healthcare costs and utilization associated with acne drugs	Retrospective cohort, following patients with acne for 90 days after first acne prescription; MPR	USA (8 states)	People with acne on national healthcare claims database, index date defined as date on which prescription for an acne-related drug first filled	24 438 eligible patients were included. 44.4% male, 55.6% female; 89.4% aged 0–17, 6.1% aged 18–25; 41% White, 50% Black, 3% Hispanic, 6% other	NR. Although some authors declare conflicts on other papers
Turan 2020^35^	To test whether acne-related emotional impairment is associated with medication adherence and psychopathology symptoms	Self-report; cross-sectional case–control	Turkey	Adolescents with acne and age- and gender-matched controls attending outpatient dermatology, Izmir, Turkey. Control participants were individuals referred during the study period for complaints other than acne	96 adolescents with acne and 100 control participants, among patients GAGS scores (severity) were a mean of 15.78 (SD 6.42) where mild = 1–18 points, moderate = 19–30 points, severe = 31–38 and very severe = 39–44. Patient gender: 46 (47.9%) male and 50 (52.1%) female. Control participant gender: 43 (43%) males/boys and 57 (57%) women/girls. Average age of patients was 15.22 (SD 1.43) years; average age of control participants 15.01 (SD 1.49)	NR
Vilar 2015^36^	To compare QoL, self-esteem and other psychosocial variables among adolescents with and without acne, and between levels of severity	Self-report; cross-sectional questionnaire survey and clinical evaluation; self-directed questionnaire including demographics, QoL and self-esteem scale	Brazil	High school students at a public school, not clear how sampled. Most participants had acne, age range not stated but all high-school students	355, of whom 317 had acne (89.3%); Total sample female 53% and male 47% (of 355); mean age 16 years (SD 1.1)	None
Zaghloul 2005^37^	To assess patient compliance in acne vulgaris and explore disease-related and social factors that influence compliance	Questionnaire and examination at baseline and 3 months later (before and after study); MPR	UK	Consecutive patients (age range 12–48 years), with diagnosis of acne and different degrees of severity	687 patients fulfilled the inclusion criteria. Of these, 403 patients (59%) attended the clinic for the second appointment at the correct time and completed both interviews. The 284 patients (41%) who could not attend the second appointment were no different in terms of disease activity and DLQI results; 182 female (45%) and 221 male (55%) participants; mean age 23.9 (SD 8.8) years (range 12–48); mean acne grade 1.42 (face, 1.05; back, 0.38; chest, 0.13) (range 0.25–4.25) on Leeds revised acne grading system; marital status, employment status and other variables reported in Table 1	No conflicts declared. No funding statement
Alnaim 2023^38^	To evaluate perceptions and extent of self‑medication and assess knowledge and patterns of self‑medication for acne	Cross-sectional survey	Saudi Arabia	University students ‘purposively sampled based on their knowledge and use of self‑medication in acne’	519 participants, of whom 476 (91.7%) reported current or past acne; 97 (17.9%) male; 422 (77.9%) female; 346 (63.8%) aged 18–21 years; 160 (29.5%) aged 22–25 years; 13 (2.4%) aged >25 years	Research Center of the Female Scientific and Medical Colleges, King Saud University
Agrawal 2025^39^	To evaluate the association between cost-related nonadherence and sex at birth among adults with acne	Cross-sectional analysis of electronic health record data	USA	People enrolled in All of Us Research Program, providing electronic health record data, sample filtered for diagnostic code for acne and who had prescription for acne within 90 days of diagnosis	5237 in final sample of whom 816 male and 4421 female. Male median age 52 years (IQR 37.8–69); Female median 45 years (IQR 35.0-59.00). 73.4% men and 74.3% women non-Hispanic White; 9% men and 9.4% women Hispanic/Latino; 8.5% men and 9.5% women non-Hispanic Black; 5.2% men and 2.5% women non-Hispanic Asian	All of Us Research Programme funded by NIH
Ling 2023^40^	To determine the effectiveness of medical education and counselling on treatment adherence and disease severity	Nonrandomized interventional study with age- and treatment-matched control participants	Malaysia	Patients with acne attending tertiary dermatology clinic	100 participants (50 interventions and 50 controls). 20% participants in intervention group had severe acne, 50% moderate acne and 30% mild acne. Mean age 22.6 years (no further details)	Not stated

BPO, benzoyl peroxide; CLDQI, Children’s Dermatology Life Quality Index; DLQI, Dermatology Life Quality Index; GAGS, Global Acne Grading System; IGA; Investigator's Global Assessment; IQR, interquartile range; MPR, ­medication possession ratio; NIH, National Institutes of Health; NR, not reported; QoL, quality of life.

Assessment of study quality (MMAT) identified a lack of methodological detail in several studies, resulting in a quality criterion of ‘unclear’. Details of methodological quality criteria scores are shown in Tables 2 and 3.

**Table 2:** Mixed Methods Appraisal Tool methodological quality criteria for quantitative nonrandomized studies (e.g. nonrandomized trials, cohort studies, case–control studies and cross-sectional analytic studies)

Paper	3.1. Are the participants representative of the target population?	3.2. Are measurements appropriate regarding both the outcome and intervention (or exposure)?	3.3. Are there complete outcome data?	3.4. Are the confounders accounted for in the design and analysis?	3.5. During the study period, is the intervention administered (or exposure occurred) as intended?
Agrawal 2025^39^	Y	U	Y	U	Y
Anderson 2015^17^	Y	Y	Y	Y	Y
Cook-Bolden 2006^19^	U	U	U	U	Y
de Lucas 2015^20^	U	Y	Y	N	Y
Gollnick 2015^23^	Y	Y	Y	Y	Y
Gollnick 2015^24^	Y	Y	Y	Y	Y
Hester 2016^16^	Y	Y	Y	Y	Y
Jones-Caballero 2008^26^	Y	Y	U	U	Y
Hoai 2021^27^	Y	Y	Y	Y	Y
Ling 2023^40^	N	Y	U	U	U
Tan 2013^15^	Y	Y	Y	Y	Y
Turan 2020^35^	U	Y	Y	U	Y
Zaghloul 2005^37^	Y	U	N	U	Y

N, no; U, unclear; Y, yes.

**Table 3:** Mixed Methods Appraisal Tool methodological quality criteria for quantitative descriptive studies (e.g. descriptive cross-sectional study, survey, ecological study)

Paper	4.1. Is the sampling strategy relevant to address the research question?	4.2. Is the sample representative of the target population?	4.3. Are the measurements appropriate?	4.4. Is the risk of nonresponse bias low?	4.5. Is the statistical analysis appropriate to answer the research question?
Alnaim 2023^38^	Y	U	N	U	Y
Balkrishnan 2006^18^	Y	Y	Y	Y	U
Dréno 2010^21^	U	U	Y	U	Y
Feldman 2011^22^	Y	U	U	U	Y
Hayran 2021^25^	U	U	U	U	Y
Liu 2022^28^	U	Y	Y	N	Y
Miyachi 2011^29^	Y	Y	Y	U	Y
Rapp 2004^30^	U	Y	Y	U	Y
Rendon 2015^31^	Y	Y	Y	Y	Y
Salamzadeh 2020^32^	U	N	Y	U	Y
Sevimli Dikicier 2019^33^	U	Y	Y	U	Y
Tan 2009^34^	U	N	Y	Y	Y
Vilar 2015^36^	U	U	Y	U	U

N, no; U, unknown (can’t tell); Y, yes.

### How is treatment adherence for acne measured?

Twenty-one studies based their measure of adherence on self-report, three papers used medication possession ratio (the number of days for which medication was dispensed divided by the number of days for which medication was needed during the study period), three objectively assessed drug refills (repeat prescriptions), one assessed pill counts and tube weight, and one carried out a drug survival analysis based on database data. Four studies used more than one method of assessment^15,19,37,40^ and in some papers the description of how adherence was assessed was not clear (see Table 4).

**Table 4:** Key findings related to adherence of included studies

Paper	How was adherence measured	Time period assessed (or cross-sectional)	Treatments assessed for adherence	Prevalence of treatment adherence	Assessed associations with adherence	Reasons given by participants for nonadherence
Anderson 2015^17^	Self-reported filling of prescriptions	Cross-sectional	Oral and topical treatments prescribed for acne	105/143 (73%) filled all prescriptions	Univariate and multivariate: Number of prescriptions Treatment type Gender Age	NR
					
					
					
					
Balkrishnan 2006^18^	Database study: annual acne-related drug refills	Cross-sectional	Oral and topical treatments prescribed for acne	Mean number of acne-related drug refills annually 2.07 (SEM 0.17)	Multivariate: Health status Health insurance	NR
Cook-Bolden 2006^19^	Self-report data and number of drug refills	6 weeks and 12 weeks	Adapalene gel 0.1% plus other acne treatments (unclear)	At week 12, self-reported adherence rates were 88.3% and 87.0%, respectively, for initial combination and add-on therapy groups. Adherence, measured by filled prescriptions at week 12, were 80.3% and 80.4%, respectively. Only percentages reported, no numbers	NR	NR
de Lucas 2015^20^	Poor adherence if at least one answer on ECOB was different from expected	1 month and 3 months	Topical retinoids or antiseptics and/or systemic antibiotics	Good adherence to acne treatment in 66.3% at 3 months. Only percentages reported, no numbers	Univariate: Use of adjuvant treatments	NR
Dréno 2010^21^	Poor adherence if at least one answer on ECOB was different from expected	Cross-sectional	Oral and topical acne treatments	Overall adherence 50% (of 3339), with regional variations of Americas 43% (of 952), Asia 48% (of 1191) and Europe 58% (of 1196). Only percentages and denominators reported	Multivariate: Age Side effects Clinical improvement Patient satisfaction Treatment type Knowledge Consultation with primary care physician	NR
Feldman 2011^22^	Self-report, asking whether stopped using the product from time to time or stopped using the product permanently	Cross-sectional	Topical clindamycin–BPO	32% used treatment less often than recommended; 10% stopped use altogether. Denominator 200, if no missing data presumed	Univariate: Side effects	NR
Gollnick 2015^23^	Questionnaire: poor adherence if at least one answer on ECOB was different from expected	3 months	Topical adapalene–BPO	Adherence was considered good in 63.2% of patients (denominator 2780)	Multivariate: Age Gender Smoking Use of moisturizer Side effects Clinical improvement	NR
Gollnick 2015^24^	Questionnaire: poor adherence if at least one answer on ECOB was different from expected	9 months	Topical adapalene–BPO alone or in combination with other drugs	Adherence was considered good for 83.9% of patients at 9 months (denominator 3977)	Univariate: Clinical improvement Acne-related QoL (CADI)	NR
Hayran 2021^25^	Self-report: 5-point Likert scale (from 5 = extremely adherent to 1 = nonadherent). Adherence defined as 5 or 4 points on Likert scale. Nonadherence defined as 3–1 points	Cross-sectional	Oral and topical acne treatments	35.6% (178/500) were adherent to treatments (Likert scores 4–5)	Univariate and multivariate: Age Education Severity Treatment duration Treatment type Treatment satisfaction Side effects	119/500 patients discontinued treatment: 48/119 (40.3%) stopped because they observed rapid improvement and 69/119 (58.0%) stopped because of aggravation of acne or side effects
Hester 2016^16^	MPR, dichotomized to adherent (≥0.8) or non­adherent (<0.8)	90 days	Oral and topical acne treatments	Overall MPR ≥0.8 in 12% (106 + 2299)/2860 + 17 179): 3.7% (106/2860) in children and 13.4% (2299/17 179) in adolescents. MPR mean (SD) 0.22 (0.21) in children and 0.37 (0.30) in adolescents	Multivariate: Age Gender Ethnicity Comorbidity index Treatment type Whether refill obtained	NR
Jones-Caballero 2008^26^	Self-report: asked patients how often they had been using treatment, using a 4-item scale (every day, almost every day, sometimes or almost never)	12 weeks	Topical 4% erythromycin + 0.2% zinc combination preparation	Topical treatment was reportedly used ‘every day’ by 934/1628 (57.4%) patients. 631/1628 patients (38.8%) used it ‘almost every day’. ‘Sometimes’ and ‘almost never’ were reported by 57 (3.5%) and 6 (0.4%) patients, respectively	Univariate: Age Gender Employment status	630/1628 (38.7%) of patients answered this question. Reasons given for nonadherence: ‘forgetting’ in 65.9% of cases, ‘lack of time’ in 15.3%, ‘boring’ in 10.7%, ‘because of side effects’ in 4.4%, and ‘other reasons’ in 3.6%
Hoai 2021^27^	Drug survival analysis	NR	Topical treatments prescribed for acne	Overall median treatment duration of all drugs was 2 months	NR	Reasons given for discontinuing treatment: ineffectiveness 52%, controlled acne 9%, side effects (9%), combination of side effects and ineffectiveness (3%) and other reasons (1%)
Liu 2022^28^	Self-report: ‘What is the shortest duration for which you have continuously used prescribed topical medication?’	Cross-sectional	75.8% of participants with acne had tried topical treatments. Survey did not ask about oral medication	When asked ‘What is the shortest duration for which you have continuously used prescribed topical medication?’ 62.9% (105/167) reported a period of less than 1 month; only 4.8% (8/167) had used prescribed topical medication for over 6 months	Univariate: Gender Academic major Acne duration Acne severity	Reasons given for discontinuing treatment: medications take too long to take effect [44.1% (71/161)]; medications have no effect [18.0%; 29/161)]; either forgot or too busy to use medication [23.6% (38/161)]; others included acne had already improved, costs, side effects
Miyachi 2011^29^	Questionnaire: poor adherence if at least one answer on ECOB was different from expected	Cross-sectional	Oral and topical acne treatments	Good adherence in 76% (no *N*). Adherence to topical medication likely to be good in 48% (of 123). Among those taking combination therapies, adherence to the topical portion of therapy was good in 51% (of 275). The likelihood of good adherence to oral medication was lower, both when it was administered alone [7% (of 30)] and when given as part of a combination regimen [14% (of 275)]	Univariate: Treatment regimen (alone or part of combination) Treatment satisfaction DLQI score Use of OTC topical antibacterials Side effects	NR
Rapp 2004^30^	Self-report: single item: ‘In the past week, how much of the time have you followed the recommendations for treating or caring for your acne?’ Rated from 1 = ‘not at all’ to 5 = ‘all of the time’. Adherence defined as reporting adherent at least some of the time (≥3)	Cross-sectional	‘Recommendations for treating or caring for your acne’	Overall, 76.6% (of 479) were adherent to medication	Multivariate: Gender Ethnicity Global QoL Skin-related QoL Treatment satisfaction Trait anger	NR
Rendon 2015^31^	Self-report: number of days of treatment use in past 4 weeks	3 months; cross-sectional?	OTC, prescription and alternative (nonmedication) acne treatments	Mean (SD) number of days treatment use in the past 3 months reported by ethnicity only: White 57 (32.4) days; Black 42.7 (33.5) days; Hispanic: 43.2 (32.9) days; Asian/other: 46.9 (37.2) days	Univariate: Ethnicity	NR
			
Salamzadeh 2020^32^	Questionnaire: poor adherence if at least one answer on ECOB was different from expected	Cross-sectional	Oral and topical acne treatments	Topical treatment adherence 19.5%, systemic treatment adherence 28.8%, total treatment adherence 15% (30/200)	Multivariate: Unclear which variables were included in multivariate analyses	NR
					
Sevimli Dikicier 2019^33^	Self-report: ‘based on ECOB’	Cross-sectional	Participants had been prescribed topical treatment for acne in past 6 months	Main adherence finding: ‘Of the 250 patients, 114 (45.6%) discontinued therapy before the scheduled time, indicating an adherence rate of 54.4%.’ ECOB findings not reported	Univariate: Acne severity	Reasons for discontinuation: unresponsiveness (62.3%) and side effects (37.7%)
Tan 2009^34^	Self-report: ‘How frequently did you use acne creams, lotions, or gels prescribed by your dermatologist at your last visit?’ Only one response permitted, with options: did not use at all (0%), 1 to 24%, 25–49%, 50–74%, 75–99%, and 100% as recommended. Adherence defined as low for the range 0–74%, medium for 75–99%, and high for 100%	Cross-sectional, asking about previous 2 months	Topical acne treatments	24% high adherence (used 100% as recommended); 49% medium adherence (used 75–99% as recommended); 26% low adherence (used 0–74% as recommended). Adherence responses obtained for 152 (but no numerators given, only percentages)	Univariate with some ‘partialling out’: Sociodemographics Acne site and severity Acne QoL Number of treatments prescribed	Reasons for low adherence were given by 30 people most commonly reported were side effects 28%, forgot to use 25%
			
Tan 2013^15^	Database study: MPR and refills. Categorized MPRs ≥0.8 as adherent and MPRs <0.8 as not adherent	90 days following index date (i.e. date on which a prescription for an acne-related drug was first filled)	Oral and topical acne treatments	Average MPR 0.34. 11.7% (2868/24 438) were categorized as adherent	Multivariate: Age Gender Ethnicity Comorbidity Treatment type Refills	NR
					Analyses of adherence incompletely presented: focus is more on healthcare costs	
Turan 2020^35^	Self-report: (MMAS: validated Turkish language version)	12 weeks (cross-sectional case–control)	Oral and topical treatments for acne	High adherence 3 (6%), medium adherence 18 (36%) and low adherence 29 (58%) for oral medication. Text suggests 46 in oral medication group but must be some missing data as percentages do not relate to 46. The percentages of high, medium and low adherence were 8 (17.4%), 26 (56.5%) and 12 (26.1%) for topical medication, respectively. Text suggests 50 in topical medication group but must be some missing data as percentages do not relate to 50	Univariate: Acne severity Strengths and Difficulties Questionnaire Difficulties in Emotion Regulation Scale	NR
Vilar 2015^36^	Self-report: unclear what questions were asked or how nonadherence was judged	Cross-sectional	Any, not stated	Among those who consulted a dermatologist, half did not follow treatment as directed, due to financial conditions	NR	Main reason was financial conditions (no data shown)
Zaghloul 2005^37^	Both MPR × 100% (pill count/weight) and interview methods	3 months questionnaire and examination at baseline and 3 months later (before and after study)	Prescribed oral and topical treatments for acne	Mean MPR was 64.7% (SD 24%) (range 0–111%). The mean interview medication adherence in the study was 93.9% (SD 5%) (range 85–100%)	Univariate: Gender Marital status Treatment type Prescription payments DLQI Smoking Alcohol	The most common reasons given by patients for nonadherence were given as being ‘fed up, forgetful or too busy’
Alnaim 2023^38^	If currently using prescribed or self-medicating treatment, how often do you use it? Always/Occasionally/Rarely	Cross-sectional	All acne treatments	Always use it 17.9% for men and 26.4% for women. Occasionally46.4% men and 55.4% women. Rarely 35.7% men and 18.2% women	NR	NR
Agrawal 2025^39^	Seven CRMA survey questions	Cross-sectional	All acne treatments	NR	Multivariate analysis shows women more likely to experience cost-related medication nonadherence, even after adjusting for income, race, ethnicity, age, BMI, comorbidity, education, insurance status and medication prescribed	NR
Ling 2023^40^	Medication adherence was assessed in weeks 0, 6 and 12 using tube weighing/pill counting and ECOB questionnaire	12 weeks	5% benzoyl peroxide gel, 0.05% tretinoin cream, oral doxycycline or oral isotretinoin based on acne severity	Tube weighing/pill count showed 64.5% adherence to 5% benzoyl peroxide gel; 51.9% adherence to 0.05% tretinoin cream; 94.3% adherence to oral antibiotics; 96.1% adherence to oral isotretinoin. ECOB scores were good for 81.0% of participants on topical treatment and 41.9% of participants on oral treatment	Patients have better adherence to topical medicine with effective disease education and treatment counselling. However, it did not improve adherence to oral medication	Forgetfulness (54%), busy lifestyle (41%), little knowledge about acne (26%) and adverse effects from topical medication (12%) included skin burning, stinging, redness and dryness

BMI, body mass index; BPO, benzoyl peroxide; CADI, Cardiff Acne Disability Index; CRMA, cost-related medication adherence; DLQI, Dermatology Life Quality Index; ECOB, Élaboration d’un outil d’évaluation de l’observance des traitements médicamenteux;^41^ MMAS, Morisky Medication Adherence Scale; MPR, medication possession ratio (the ratio of the number of days for which medication was dispensed divided by the number of days for which medication was needed during study period; this method assumes that, if a prescription was filled, the drugs were taken or applied.); NR, not reported; OTC, over the counter (i.e. acne prescription bought by participant, not prescribed by clinician); QoL, quality of life.

#### Self-report

Assessment of acne treatment adherence using self-report included the use of validated scales (Morisky Medication Adherence Scale, which focuses on recent medication-taking; ‘implementation’ adherence),^35^ and some other bespoke questionnaires reporting current adherence (‘implementation’ adherence).^25,26,30,31,34,38^ Some focused on self-report of whether refills (or repeat prescriptions) had not been collected (‘discontinuation’ adherence),^17^ and some focused on a combination of implementation and discontinuation.^22,28^ One study focused on cost barriers to adherence.^39^ In some studies, little detail was given of the self-report questions.^19,36^ For several studies including self-­reported treatment adherence, this was not really their main focus but was assessed in relation to treatment preference or treatment satisfaction (see Table 4 for details).

Eight studies reported on versions of the ‘Élaboration d’un outil d’évaluation de l’observance des traitements médicamenteux’ (ECOB), developed with support from the pharmaceutical industry as a mini-questionnaire to assess adherence, with separate questions for oral vs. topical acne treatments.^41^ Good adherence is defined as answering all four questions of the ECOB as expected (i.e. if one or more questions differs from the expected answer then adherence is considered to be ‘poor’). Question 1 of the ECOB asks ‘Do you remember the name of the (last) drug(s) you took?’ Questions 2, 3 and 4 ask about tolerability, stopping the drug and whether the drug has been useful.^41^ It can be seen from some studies that used the ECOB that the answer that most frequently differs from expected is Question 1.^23,24,29^ However, some studies did not report the responses to individual items within the ECOB^20,21,33^ so this cannot always be assessed. Some authors^21^ stated that if participants recalled the colour of the treatment packaging then it was accepted as a correct answer to Question 1, whereas others did not mention this.

#### Medication possession ratio or ‘refills’

The medication possession ratio (MPR) (the ratio of the number of days for which medication was dispensed divided by the number of days for which medication was needed during the study period) assumes that, if a prescription was obtained, the drugs were taken (oral treatments) or applied (topical treatments). Clearly for topical treatments this can be a more problematic calculation due to variations in skin coverage (face only or face and torso). One study attempted to assess the approximate surface area of acne in relation to the weight of topical treatment used,^37^ whereas others did not include data on surface area.^15,16^ Similarly, these two related studies dichotomized the MPR to categorize patients as adherent (≥0.8) or nonadherent (<0.8), although it is not described how this cutoff was chosen.^15,16^ Others argue for viewing the MPR as a continuous measure of adherence to avoid arbitrarily characterizing participants as ‘compliers’ or ‘noncompliers’.^37^

Three papers assessed adherence based on the number of prescription ‘refills’ over a given time period.^15,18,19^ ‘Refills’ occur where the patient obtains additional prescribed medication for ongoing treatment without further consultation, otherwise known as ‘repeat prescription’ or ‘repeat dispensing’. A drug survival analysis in Belgium looked at how long patients remained on acne treatments by reviewing medical records.^27^

#### Objective assessment

One study carried out among 100 people with acne in a tertiary dermatology clinic used both objective assessment by pill count (oral medication) and tube weighing (topical treatments), as well as self-report with the ECOB questionnaire.^40^

### What is the prevalence of treatment adherence in acne?

The estimated prevalence of acne treatment adherence varied widely from 6% to 84% (see Table 4). Primary nonadherence, or noninitiation of treatment, appears to be a common form of nonadherence, with one study finding that 27% of participants reported not filling all prescriptions prescribed at their previous clinical appointment for acne.^17^ Most other studies were less clear on whether adherence rates related to initiation, implementation or discontinuation.

The study reporting the lowest treatment adherence rates (6% adherence to oral acne medication and 17% to topical acne treatments) included only 96 young people aged 12–17 years with acne and was the only study to use the Morisky Medication Adherence Scale (implementation), so comparisons are limited.^35^ Among studies reporting ECOB scores (implementation and discontinuation), reported adherence ranged from 15% to 83.9%. The study reporting the lowest overall treatment adherence (15%) was from Iran and the authors suggest this may have been related to greater reliance on traditional or alternative medicine in this population.^32^ Larger cross-sectional surveys that used the ECOB to look at oral and topical treatments found adherence to be around 50%,^21^ whereas in the context of a cohort study, treatment adherence was much higher at 83.9%.^24^ Few studies allow international comparisons other than the large study by Dréno *et al*., who estimated the risk of poor adherence as follows: Americas 43%, Asia 48% and Europe 58%.^21^

A drug survival analysis (discontinuation nonadherence) carried out in over 1000 patients in 1 hospital and 3 private practices in Belgium reported a median duration of all topical acne treatments of 2 months, with only 50% continuing treatment at 3 months.^27^ A large US database study showed that the mean number of acne-related drug refills annually was 2.07 (SEM 0.17), but in the absence of information on the volume of each prescription, these findings are difficult to interpret.^18^

For studies where treatment adherence was assessed using MPR, the prevalence of good treatment adherence was estimated at approximately 12% for studies where MPR was dichotomized to adherent (≥0.8) or nonadherent (<0.8).^15,16^ Looking at the reported mean MPR, rather than dichotomized outcome, this was 0.34 among US Medicaid enrollees, suggesting that most patients took their treatment less than half of the time.^15^ In a UK dermatology setting the mean MPR was found to be 0.65, suggesting that most patients took their treatment most of the time.^37^

A cross-sectional survey of college students in China asked, ‘What is the shortest duration for which you have continuously used prescribed topical medication?’ and found that 63% of those using acne treatments reported a period of <1 month, suggesting still greater rates of discontinuation, although the methodology and population differed from other studies.^28^

The one study that weighed topical treatment used at 12 weeks found adherence rate of 64.5% of expected for 5% BPO gel and 51.9% of expected for 0.05% tretinoin cream, based on estimating the acne surface area with one ‘hand area’ requiring half a fingertip unit, or 0.25 g, topical treatment: for oral antibiotics, pill counts showed 94.3% expected treatment use.^40^

### Factors associated with treatment adherence in acne

Factors examined for possible associations with treatment adherence in acne are shown in Table 4. Many of the variables associated with treatment adherence are themselves correlated (e.g. acne severity with treatment type, or healthcare provider, health insurance with geography), so we therefore focused on studies reporting multivariate analyses.

Of the studies that assessed the association between age and treatment adherence, two large studies found that younger age (<15 years or <12 years) was associated with lower adherence.^16,42^ Others found no difference in adherence in relation to age, but these were studies with smaller or more select samples, or only included participants aged ≥18 years.^17,23,25^ Most studies found no difference in treatment adherence on the basis of gender when included as part of a multivariate analysis,^16,17,21,23,25^ with the exception of one smaller study where young men were found to be less adherent.^30^ Conversely, the study that addressed cost barriers to treatment adherence found these to be greater among individuals who were assigned female sex at birth.^39^

There were conflicting findings regarding whether treatment adherence is more likely with topical or oral acne treatments (oral acne treatments other than isotretinoin). In one large survey using the ECOB, adherence to topical treatments was found to be better than to oral treatments for people who had been prescribed both.^21^ A large US database study using MPR found good adherence to the contraceptive pill and topical retinoids but low adherence to oral antibiotics amongst Medicaid enrollees.^15^ In a study on the same database, but only including people aged <18 years, good adherence to topical retinoids was found.^16^ A large survey in Turkey also found no difference between self-reported adherence to topical treatments and oral antibiotics.^25^ One US study found that participants were less likely to report filling prescriptions for topical ­retinoids than other acne treatments.^17^ Interestingly, the one study that used both objective and subjective assessments of treatment adherence reported a discrepancy between adherence to oral acne treatments measured using an objective assessment (pill count) and self-report (ECOB).^40^ Adherence was better in the objective assessment and the authors attribute this to the wording of the ECOB: ‘Question 3 asks if the subject ever stopped taking topical drugs because they thought it would do more harm than good.’ Where participants missed topical medication this was not thought to be due to the harmful effects of treatment, whereas participants taking oral treatments would answer ‘yes’ to this question even if they had only missed 1 day in 12 weeks.^40^

### Reasons given for nonadherence

Eight studies reported reasons that participants gave for non­adherence to acne treatments.^25–28,33,34,36,37^ Although the nature of the questions and the settings vary, forgetfulness, perceived ineffectiveness, side effects and treatment costs were commonly cited (see Table 4). One Turkish study (*n* = 500) reported that 40% of patients stopped because they observed rapid improvement.^25^ However, a larger Belgian study (*n* = 1029) found that only 9% of patients reported stopping due to controlled acne, with 52% citing perceived ineffectiveness and 9% citing side effects.^27^

## Discussion

Estimates of treatment adherence in acne vary widely, with variation relating to study design and population, whether the focus was on initiation, implementation or discontinuation treatment non­adherence, which treatments were being assessed and how adherence was assessed. Contrary to popular belief, there is little ­evidence for lower adherence to topical treatments than oral treatments, although this may be related to the heterogeneity by which adherence is assessed, as the one study that used both objective measures and self-report found higher adherence to oral treatment by pill count but lower adherence when assessed using questionnaire.^40^ In common with findings from other reviews, younger age (<15 years) is associated with low treatment adherence for acne, but there is no consistent evidence for differences between men/boys and women/girls. Common reasons given for discontinuing treatment are cost issues, side effects, perceived ineffectiveness or, conversely, rapid improvement, highlighting one of the challenges of assessing treatment adherence for this condition, in that nonadherence can be related to resolution of acne.

The 2014 systematic review of RCTs on treatment adherence in acne found a similarly broad range of adherence prevalence estimates ranging from 7% to 98%, but commented on methodological weaknesses and that clinical trial participants are more likely to be adherent than other populations.^10^ They found that low adherence was more common among younger patients and no significant difference between self-reported adherence to topical vs. oral treatments.^10^ Our review goes further in systematically examining associations with nonadherence and using observational studies, where estimates of adherence are more likely to reflect everyday use. Our findings regarding nonadherence are in line with previous reviews of quantitative and qualitative literature.^11,43^ The review by Lo *et al*.^11^ highlights that access to treatments is an important factor in dermatological treatment adherence, which may account for some of the variations seen in this study. For instance, the high cost and insurance approval process for topical retinoids in the US contrasts with the UK, where prescriptions incur no cost for people on a low income and for most people aged <19 years, and where a cost is incurred, this is a fixed rate. Furthermore, the availability of treatments available over the counter without a prescription varies between countries.

More robust findings probably relate to the MPR taking the affected surface area into account, where adherence was estimated as 65%.^37^ Similarly, drug survival analysis, showing that 50% of patients continue to use treatments at 3 months, is not dissimilar.^27^ These findings reflect a large study of general practitioner prescribing for acne in the UK, which showed that after an initial prescription for acne medications, 60% of patients were prescribed no further acne medications.^44^

This was a rigorous review, taking a methodical approach to assessing treatment adherence and the first, to our knowledge, to review factors associated with nonadherence.

While some studies asked about acne treatments obtained without a prescription,^17,31,38^ most focused on prescribed treatments. This is a limitation of our study as it limits the population to people who have consulted rather than people who self-manage their acne. It is possible that adherence to treatments purchased without prescription may be different, although there are likely to be large international variations due to differences in the availability of acne treatments without prescription in different territories.

As with any systematic review, the conclusions are limited by the number and limitations of the papers identified. One limitation is the different approaches to assessing adherence identified in the papers in this review, illuminating different aspects of treatment adherence such as initiation, implementation and discontinuation,^9^ and measures based on ‘refills’ may reflect noninitiation while self-report may be more appropriate for assessing implementation. Many of the studies reported on could have reflected more explicitly on these categories, which are embedded within reporting guidelines for studies on medication adherence.^45^

Another limitation is that most of the studies in the review had links with pharmaceutical organizations, which may raise questions. Relying on recall of the name of a treatment when assessing adherence makes it difficult to draw conclusions from these studies using the ECOB, as this method of assessment may favour products with more prominent branding or easier names, rather than how regularly treatments are used.

While we initially aimed to carry out a mixed-methods study including all quantitative and qualitative studies on treatment adherence in acne, we have focused on observational quantitative data here, in order to allow a full exploration of the data. The qualitative evidence will be reported separately and will allow greater exploration of people’s perceptions and experiences around acne treatments. A systematic review of randomized trials will examine which approaches to enhancing adherence have or have not been shown to be effective.

Our findings highlight the need for future research to establish more standardized measures for self-reporting treatment adherence in acne and explore different aspects of treatment adherence such as initiation, implementation and discontinuation. Our findings also point to the clinical importance of addressing treatment adherence in acne, and that potential solutions should be aimed at mitigating against side effects, ensuring that people with acne understand that treatments take several weeks of regular use to take effect, offering tools to support adherence and offering proactive review.

## Supplementary Material

vzag084_Supplementary_Data

## Data Availability

The data underlying this article will be shared on reasonable request to the corresponding author.
