# Supplementary material for: Treatment adherence in acne vulgaris: systematic review and narrative synthesis
Source: Skin Health Dis. 2026 Jul 31;6(5):690–711. doi: 10.1093/skinhd/vzag084 (PMC13623435; doi:10.1093/skinhd/vzag084)
Supplement: Supplementary file 1 [file vzag084_supplementary_data.docx]

# Appendix S1 – search strategies

MEDLINE (Ovid) 1946 to 24^th^ March 2025

| **Search number** | **Query** |
| --- | --- |
| 1 | exp Acne Vulgaris/ |
| 2 | Acne.mp. |
| 3 | 1 or 2 |
| 4 | Randomized Controlled Trials as Topic/ |
| 5 | controlled clinical trial.pt. |
| 6 | randomized controlled trial.pt. |
| 7 | (randomi#ed or randomi#ation).ti,ab. |
| 8 | randomly.ab. |
| 9 | trial.ti,ab. |
| 10 | groups.ab. |
| 11 | (control* adj3 (trial* or study or studies)).ti,ab. |
| 12 | clinical study.ti,ab. |
| 13 | (quasi adj (experimental or random$)).ti,ab. |
| 14 | 4 or 5 or 6 or 7 or 8 or 9 or 10 or 11 or 12 or 13 |
| 15 | (questionnaire* or survey*).mp. |
| 16 | EPIDEMIOLOGIC STUDIES/ |
| 17 | exp CASE CONTROL STUDIES/ |
| 18 | exp COHORT STUDIES/ |
| 19 | Case control.tw. |
| 20 | (cohort adj (study or studies)).tw. |
| 21 | Cohort analy$.tw. |
| 22 | (Follow up adj (study or studies)).tw. |
| 23 | (observational adj (study or studies)).tw. |
| 24 | Longitudinal.tw. |
| 25 | Retrospective.tw. |
| 26 | Prospective.tw. |
| 27 | Cross sectional.tw. |
| 28 | CROSS-SECTIONAL STUDIES/ |
| 29 | mixed method*.tw. |
| 30 | 15 or 16 or 17 or 18 or 19 or 20 or 21 or 22 or 23 or 24 or 25 or 26 or 27 or 28 or 29 |
| 31 | (factor* or predict* or associat* or correlat* or moderator* or mediator* or determin*).tw. |
| 32 | process evaluation.ti,ab,kw. |
| 33 | implementation evaluation.tw. |
| 34 | 31 or 32 or 33 |
| 35 | 14 or 30 or 34 |
| 36 | exp Patient Compliance/ |
| 37 | exp Treatment Failure/ |
| 38 | exp "Treatment Adherence and Compliance"/ |
| 39 | HEALTH KNOWLEDGE, ATTITUDES, PRACTICE/ |
| 40 | Help seeking behavior/ |
| 41 | health care costs/ |
| 42 | (acceptance or acceptability or adherence or nonadherence or non-adherence or compliance or noncompliance or non-compliance or concordance or nonconcordance or non-concordance or persever* or belief* or attitude* or satisfact* or preference* or perspective* or perception*).ti,ab,kf. |
| 43 | (cease or cessation or continuation or discontinu* or dropout or drop out or interrupt* or taper* or reduce or drug holiday or stop or stopping or withdraw* or terminat*).ti,kf,hw. |
| 44 | (stop* using or stop* taking or stop* treatment).ti,ab,kf. |
| 45 | (help-seeking or seek* help or medical consult* or medical advice or treatment advice or treatment seek* or Seek treatment or advice or knowledge or information).ti,ab,kf. |
| 46 | (cost* or out-of-pocket or price* or expenditure* or budget* or willingness-to-pay or fee or fees or charge*).ti,ab,kf. |
| 47 | 36 or 37 or 38 or 39 or 40 or 41 or 42 or 43 or 44 or 45 or 46 |
| 48 | (patient* or child* or teenage* or adolescen* or young or consumer* or clinician* or physician* or GP or GPs or healthcare professional* or consultant or nurse* or pharmacist* or practitioner* or parent* or caregiver* or care giver or mother or father or paternal or maternal or guardian).ti,ab,kf. |
| 49 | 3 and 35 and 47 and 48 |
| 50 | (cancer* or carcinoma* or tumor* or tumour* or laser* or microneedl* or photodynamic*).ti. |
| 51 | 49 not 50 |
| 52 | remove duplicates from 51 |

Embase (Ovid)1947 to 02 June 2023

| **Search number** | **Query** |
| --- | --- |
| 1 | acne.ab,ti. |
| 2 | exp acne vulgaris/ |
| 3 | 1 or 2 |
| 4 | randomized controlled trial.de. |
| 5 | randomi#ed.ti,ab. |
| 6 | randomly.ab. |
| 7 | factorial$.ti,ab. |
| 8 | (control$ adj3 (trial$ or study or studies or group$)).ti,ab. |
| 9 | (quasi adj (experimental or random$)).mp. |
| 10 | clinical study.ti,ab. |
| 11 | 4 or 5 or 6 or 7 or 8 or 9 or 10 |
| 12 | questionnaire/ |
| 13 | case control study/ |
| 14 | Longitudinal study/ |
| 15 | Retrospective study/ |
| 16 | Prospective study/ |
| 17 | Cohort analysis/ |
| 18 | (questionnaire* or survey*).mp. |
| 19 | (Cohort adj (study or studies)).mp. |
| 20 | (Case control adj (study or studies)).tw. |
| 21 | (follow up adj (study or studies)).tw. |
| 22 | (observational adj (study or studies)).tw. |
| 23 | (epidemiologic$ adj (study or studies)).tw. |
| 24 | (cross sectional adj (study or studies)).tw. |
| 25 | mixed method*.tw. |
| 26 | 12 or 13 or 14 or 15 or 16 or 17 or 18 or 19 or 20 or 21 or 22 or 23 or 24 or 25 |
| 27 | (factor* or predict* or associat* or correlat* or moderator* or mediator* or determin*).tw. |
| 28 | process evaluation.ti,ab,kw. |
| 29 | implementation evaluation.tw. |
| 30 | 27 or 28 or 29 |
| 31 | 11 or 26 or 30 |
| 32 | patient compliance/ |
| 33 | treatment failure/ |
| 34 | ATTITUDE TO HEALTH/ |
| 35 | HEALTH PERSONNEL ATTITUDE/ |
| 36 | PATIENT ATTITUDE/ or MEDICATION COMPLIANCE/ |
| 37 | help seeking behavior/ |
| 38 | health care costs/ |
| 39 | (acceptance or acceptability or adherence or nonadherence or non-adherence or compliance or noncompliance or non-compliance or concordance or nonconcordance or non-concordance or persever* or belief* or attitude* or satisfact* or preference* or perspective* or perception*).ti,ab,kf. |
| 40 | (cease or cessation or continuation or discontinu* or dropout or drop out or interrupt* or taper* or reduce or drug holiday or stop or stopping or withdraw* or terminat*).ti,kf,hw. |
| 41 | (stop* using or stop* taking or stop* treatment).ti,ab,kf. |
| 42 | (help-seeking or seek* help or medical consult* or medical advice or treatment advice or treatment seek* or Seek treatment or advice or knowledge or information).ti,ab,kf. |
| 43 | (cost* or out-of-pocket or price* or expenditure* or budget* or willingness-to-pay or fee or fees or charge*).ti,ab,kf. |
| 44 | 32 or 33 or 34 or 35 or 36 or 37 or 38 or 39 or 40 or 41 or 42 or 43 |
| 45 | (patient* or child* or teenager* or adolescen* or young or consumer* or clinician* or physician* or GP or GPs or healthcare professional* or consultant or nurse* or pharmacist* or practitioner* or parent* or caregiver* or care giver or mother or father or paternal or maternal or guardian).ti,ab,kf. |
| 46 | 3 and 31 and 44 and 45 |
| 47 | (cancer* or carcinoma* or tumor* or tumour* or laser* or microneedl* or photodynamic*).ti. |
| 48 | 46 not 47 |
| 49 | remove duplicates from 48 |

PubMed

| **Search number** | **Query** |
| --- | --- |
| 49 | #47 NOT #48 |
| 48 | ((cancer*[TIAB] or carcinoma*[TIAB] or tumor*[TIAB] or tumour*[TIAB] or laser*[TIAB] or microneedl*[TIAB] or photodynamic*[TIAB])) |
| 47 | #3 AND #33 AND #45 AND #46 |
| 46 | (patient*[TIAB] or child*[TIAB] or teenage*[TIAB] or adolescen*[TIAB] or young[TIAB] or consumer*[TIAB] or clinician*[TIAB] or physician*[TIAB] or GP[TIAB] or GPs[TIAB] or healthcare professional*[TIAB] or consultant[TIAB] or nurse*[TIAB] or pharmacist*[TIAB] or practitioner*[TIAB] or parent*[TIAB] or caregiver*[TIAB] or care giver[TIAB] or mother[TIAB] or father[TIAB] or paternal[TIAB] or maternal[TIAB] or guardian[TIAB]) |
| 45 | #34 OR #35 OR #36 OR #37 OR #38 OR #39 OR #40 OR #41 OR #42 OR #43 OR #44 |
| 44 | (cost*[TIAB] or out-of-pocket[TIAB] or price*[TIAB] or expenditure*[TIAB] or budget*[TIAB] or willingness-to-pay[TIAB] or fee[TIAB] or fees[TIAB] or charge*[TIAB]) |
| 43 | (help-seeking[TIAB] or seek* help[TIAB] or medical consult*[TIAB] or medical advice[TIAB] or treatment advice[TIAB] or treatment seek*[TIAB] or Seek treatment[TIAB] or advice[TIAB] or knowledge[TIAB] or information[TIAB]) |
| 42 | (stop* using[TIAB] or stop* taking[TIAB] or stop* treatment[TIAB]) |
| 41 | (cease[TIAB] or cessation[TIAB] or continuation[TIAB] or discontinu*[TIAB] or dropout[TIAB] or drop out[TIAB] or interrupt*[TIAB] or taper*[TIAB] or reduce[TIAB] or drug holiday[TIAB] or stop[TIAB] or stopping[TIAB] or withdraw*[TIAB] or terminat*[TIAB]) |
| 40 | (acceptance[TIAB] or acceptability[TIAB] or adherence[TIAB] or nonadherence[TIAB] or non-adherence[TIAB] or compliance[TIAB] or noncompliance[TIAB] or non-compliance[TIAB] or concordance[TIAB] or nonconcordance[TIAB] or non-concordance[TIAB] or persever*[TIAB] or belief*[TIAB] or attitude*[TIAB] or satisfact*[TIAB] or preference*[TIAB] or perspective*[TIAB] or perception*[TIAB]) |
| 39 | "health care costs"[MeSH:NOEXP] |
| 38 | "help seeking behavior"[MeSH Terms] |
| 37 | HEALTH KNOWLEDGE, ATTITUDES, PRACTICE[MESH:NOEXP] |
| 36 | "treatment adherence and compliance"[MeSH Terms] |
| 35 | "treatment failure"[MeSH Terms] |
| 34 | patient compliance[MeSH Terms] |
| 33 | #14 OR #28 OR #32 |
| 32 | #29 OR #30 OR #31 |
| 31 | implementation evaluation[TIAB] |
| 30 | process evaluation[TIAB] |
| 29 | (factor*[TW] or predict*[TW] or associat*[TW] or correlat*[TW] or moderator*[TW] or mediator*[TW] or determin*[TW]) |
| 28 | #15 OR #16 OR #17 OR #18 OR #19 OR #20 OR #21 OR #22 OR #23 OR #24 OR #25 OR #26 OR #27 |
| 27 | mixed method*[TW] |
| 26 | Cross sectional[TIAB] |
| 25 | Retrospective[TIAB] |
| 24 | Longitudinal[TIAB] |
| 23 | ((observational study[TIAB] or observational studies[TIAB])) |
| 22 | ((Follow up study[TIAB] or follow up studies[TIAB])) |
| 21 | Cohort analy*[TIAB] |
| 20 | ((cohort study[TIAB] or cohort studies[TIAB])) |
| 19 | Case control[TIAB] |
| 18 | cohort studies[MESH] |
| 17 | Case-control studies[MESH] |
| 16 | Epidemiologic studies[MESH:NOEXP] |
| 15 | (questionnaire*[ALL] or survey*[ALL]) |
| 14 | (#4 OR #5 OR #6 OR #7 OR #8 OR #9 OR #10 OR #11 OR #12 OR #13) |
| 13 | (((quasi experimental[TIAB] or quasi random*[TIAB]))) |
| 12 | clinical study[TIAB] |
| 11 | ((control* trial*[TIAB] or control* study[TIAB] or control* studies[TIAB])) |
| 10 | groups[TIAB] |
| 9 | trial[TIAB] |
| 8 | randomly[TIAB] |
| 7 | (((randomized[TIAB] or randomised[TIAB] or randomization[TIAB] or randomisation[TIAB]))) |
| 6 | randomized controlled trial[PT] |
| 5 | controlled clinical trial[PT] |
| 4 | randomized controlled trial [MESH:NOEXP] |
| 3 | (#1 OR #2) |
| 2 | Acne |
| 1 | "acne vulgaris"[MeSH Terms] |

CINAHL (EBSCOhost)

| **Search number** | **Query** |
| --- | --- |
| S41 | S39 NOT S40 |
| S40 | (TI (cancer* or carcinoma* or tumour* or tumour* or arthroplasty or laser* or microneedl* or photodynamic*) |
| S39 | S3 AND S26 AND S37 AND S38 |
| S38 | (TI(patient* or child* or teenage* or adolescen* or young or consumer* or clinician* or physician* or GP or GPs or healthcare professional* or consultant or nurse* or pharmacist* or practitioner* or parent* or caregiver* or care giver or mother or father or paternal or maternal or guardian)) OR (AB(patient* or child* or teenage* or adolescen* or young or consumer* or clinician* or physician* or GP or GPs or healthcare professional* or consultant or nurse* or pharmacist* or practitioner* or parent* or caregiver* or care giver or mother or father or paternal or maternal or guardian) OR (MW(patient* or child* or teenage* or adolescen* or young or consumer* or clinician* or physician* or GP or GPs or healthcare professional* or consultant or nurse* or pharmacist* or practitioner* or parent* or caregiver* or care giver or mother or father or paternal or maternal or guardian)) |
| S37 | S27 OR S28 OR S29 OR S30 OR S31 OR S32 OR S33 OR S34 OR S35 OR S36 |
| S36 | (TI(cost* or out-of-pocket or price* or expenditure* or budget* or willingness-to-pay or fee or fees or charge*)) OR (AB (cost* or out-of-pocket or price* or expenditure* or budget* or willingness-to-pay or fee or fees or charge*) OR (MW(cost* or out-of-pocket or price* or expenditure* or budget* or willingness-to-pay or fee or fees or charge*)) |
| S35 | (TI(help-seeking or seek* help or medical consult* or medical advice or treatment advice or treatment seek* or Seek treatment or advice or knowledge or information)) OR (AB(help-seeking or seek* help or medical consult* or medical advice or treatment advice or treatment seek* or Seek treatment or advice or knowledge or information)) OR (MW(help-seeking or seek* help or medical consult* or medical advice or treatment advice or treatment seek* or Seek treatment or advice or knowledge or information)) |
| S34 | (TI(stop* using or stop* taking or stop* treatment)) OR (AB(stop* using or stop* taking or stop* treatment)) OR (MW(stop* using or stop* taking or stop* treatment)) |
| S33 | (TI(cease or cessation or continuation or discontinu* or dropout or drop out or interrupt* or taper* or reduce or drug holiday or stop or stopping or withdraw* or terminat*)) OR (AB(cease or cessation or continuation or discontinu* or dropout or drop out or interrupt* or taper* or reduce or drug holiday or stop or stopping or withdraw* or terminat*)) OR (MW(cease or cessation or continuation or discontinu* or dropout or drop out or interrupt* or taper* or reduce or drug holiday or stop or stopping or withdraw* or terminat*)) |
| S32 | (TI(acceptance or acceptability or adherence or nonadherence or non-adherence or compliance or noncompliance or non-compliance or concordance or nonconcordance or non-concordance or persever* or belief* or attitude* or satisfact* or preference* or perspective* or perception*)) OR (AB(acceptance or acceptability or adherence or nonadherence or non-adherence or compliance or noncompliance or non-compliance or concordance or nonconcordance or non-concordance or persever* or belief* or attitude* or satisfact* or preference* or perspective* or perception*) OR (MW(acceptance or acceptability or adherence or nonadherence or non-adherence or compliance or noncompliance or non-compliance or concordance or nonconcordance or non-concordance or persever* or belief* or attitude* or satisfact* or preference* or perspective* or perception*)) |
| S31 | (MH "Health Care Costs") |
| S30 | (MH "Help Seeking Behavior") |
| S29 | (MH "Health Knowledge") |
| S28 | (MH "Attitude to Health") or (MH "Attitude to Illness") or (MH "Attitude of Health Personnel") or (MH "Patient Attitudes") |
| S27 | (MH "Patient Compliance") or (MH "Medication Compliance") or (MH "Consumer Participation") or (MH "Patient Satisfaction") or (MH "Treatment Refusal") |
| S26 | S9 OR S21 OR S25 |
| S25 | S22 OR S23 OR S24 |
| S24 | (TI(Implementation N1 evaluation)) or (AB(Implementation N1 evaluation)) or (MW(Implementation N1 evaluation)) |
| S23 | (TI(Process N1 evaluation)) or (AB(Process N1 evaluation)) or (MW(Process N1 evaluation)) |
| S22 | (TI(factor* or predict* or associat* or correlat* or moderator* or mediator* or determin*)) OR (AB(factor* or predict* or associat* or correlat* or moderator* or mediator* or determin*)) OR (MW(factor* or predict* or associat* or correlat* or moderator* or mediator* or determin*)) |
| S21 | S10 OR S11 OR S12 OR S13 OR S14 OR S15 OR S16 OR S17 OR S18 OR S19 OR S20 |
| S20 | (TI(Mixed N1 method*)) or (AB(Mixed N1 method*)) or (MW(Mixed N1 method*)) |
| S19 | (TI (cross sectional N1 (study or studies))) or (AB (cross sectional N1 (study or studies))) or (MW (cross sectional N1 (study or studies))) |
| S18 | (TI(Prospective)) or (AB(Prospective)) or (MW(Prospective)) |
| S17 | (TI(Retrospective)) or (AB(Retrospective)) or (MW(Retrospective)) |
| S16 | (TI(Longitudinal)) or (AB(Longitudinal)) or (MW(Longitudinal)) |
| S15 | (TI (observational N1 (study or studies))) or (AB (observational N1 (study or studies))) or (MW (observational N1 (study or studies))) |
| S14 | (TI (follow up N1 (study or studies))) or (AB (follow up N1 (study or studies))) or (MW (follow up N1 (study or studies))) |
| S13 | (TI (cohort N1 (study or studies))) or (AB (cohort N1 (study or studies))) or (MW (cohort N1 (study or studies))) |
| S12 | (TI (case N1 control)) or (AB (case N1 control)) or (MW (case N1 control)) |
| S11 | (TI (epidemiologic* N1 (study or studies))) or (AB (epidemiologic* N1 (study or studies))) or (MW (epidemiologic* N1 (study or studies))) |
| S10 | questionnaire* or survey* |
| S9 | S4 OR S5 OR S6 OR S7 OR S8 |
| S8 | (TI (quasi N1 (experimental OR randomi*))) or (AB (quasi N1 (experimental OR randomi*))) |
| S7 | (TI (control* N3 (trial* or study or studies))) or (AB (control* N3 (trial* or study or studies))) |
| S6 | (TI (controlled N1 trial*)) or (AB (controlled N1 trial*)) |
| S5 | (TI (randomi* control* trial*)) or (AB (randomi* control* trial*)) |
| S4 | (TI (clinic* N1 (trial* or study or studies))) OR (AB(clinic* N1 (trial* or study or studies))) |
| S3 | S1 OR S2 |
| S2 | acne |
| S1 | MH acne vulgaris |

PsycINFO (EBSCOhost)

| **Search number** | **Query** |
| --- | --- |
| S40 | S3 AND S26 AND S38 AND S39 |
| S39 | (TI(patient* or child* or teenage* or adolescen* or young or consumer* or clinician* or physician* or GP or GPs or healthcare professional* or consultant or nurse* or pharmacist* or practitioner* or parent* or caregiver* or care giver or mother or father or paternal or maternal or guardian)) OR (AB(patient* or child* or teenage* or adolescen* or young or consumer* or clinician* or physician* or GP or GPs or healthcare professional* or consultant or nurse* or pharmacist* or practitioner* or parent* or caregiver* or care giver or mother or father or paternal or maternal or guardian) OR (KW(patient* or child* or teenage* or adolescen* or young or consumer* or clinician* or physician* or GP or GPs or healthcare professional* or consultant or nurse* or pharmacist* or practitioner* or parent* or caregiver* or care giver or mother or father or paternal or maternal or guardian)) |
| S38 | S27 OR S28 OR S29 OR S30 OR S31 OR S32 OR S33 OR S34 OR S35 OR S36 OR S37 |
| S37 | (TI(cost* or out-of-pocket or price* or expenditure* or budget* or willingness-to-pay or fee or fees or charge*)) OR (AB (cost* or out-of-pocket or price* or expenditure* or budget* or willingness-to-pay or fee or fees or charge*) OR (KW(cost* or out-of-pocket or price* or expenditure* or budget* or willingness-to-pay or fee or fees or charge*)) |
| S36 | (TI(help-seeking or seek* help or medical consult* or medical advice or treatment advice or treatment seek* or Seek treatment or advice or knowledge or information)) OR (AB(help-seeking or seek* help or medical consult* or medical advice or treatment advice or treatment seek* or Seek treatment or advice or knowledge or information)) OR (KW(help-seeking or seek* help or medical consult* or medical advice or treatment advice or treatment seek* or Seek treatment or advice or knowledge or information)) |
| S35 | (TI(stop* using or stop* taking or stop* treatment)) OR (AB(stop* using or stop* taking or stop* treatment)) OR (KW(stop* using or stop* taking or stop* treatment)) |
| S34 | (TI(cease or cessation or continuation or discontinu* or dropout or drop out or interrupt* or taper* or reduce or drug holiday or stop or stopping or withdraw* or terminat*)) OR (AB(cease or cessation or continuation or discontinu* or dropout or drop out or interrupt* or taper* or reduce or drug holiday or stop or stopping or withdraw* or terminat*)) OR (KW(cease or cessation or continuation or discontinu* or dropout or drop out or interrupt* or taper* or reduce or drug holiday or stop or stopping or withdraw* or terminat*)) |
| S33 | (TI(acceptance or acceptability or adherence or nonadherence or non-adherence or compliance or noncompliance or non-compliance or concordance or nonconcordance or non-concordance or persever* or belief* or attitude* or satisfact* or preference* or perspective* or perception*)) OR (AB(acceptance or acceptability or adherence or nonadherence or non-adherence or compliance or noncompliance or non-compliance or concordance or nonconcordance or non-concordance or persever* or belief* or attitude* or satisfact* or preference* or perspective* or perception*) OR (KW(acceptance or acceptability or adherence or nonadherence or non-adherence or compliance or noncompliance or non-compliance or concordance or nonconcordance or non-concordance or persever* or belief* or attitude* or satisfact* or preference* or perspective* or perception*)) |
| S32 | DE health care costs |
| S31 | DE health care seeking behavior |
| S30 | DE "client participation" or DE "client satisfaction" or DE "treatment refusal" |
| S29 | DE "health knowledge" |
| S28 | DE "health attitudes" or DE "client attitudes" or DE "health personnel attitudes" |
| S27 | DE treatment compliance |
| S26 | S9 OR S21 OR S25 |
| S25 | S22 OR S23 OR S24 |
| S24 | (TI(Implementation N1 evaluation)) or (AB(Implementation N1 evaluation)) or (KW(Implementation N1 evaluation)) |
| S23 | (TI(Process N1 evaluation)) or (AB(Process N1 evaluation)) or (KW(Process N1 evaluation)) |
| S22 | (TI(factor* or predict* or associat* or correlat* or moderator* or mediator* or determin*)) OR (AB(factor* or predict* or associat* or correlat* or moderator* or mediator* or determin*)) OR (KW(factor* or predict* or associat* or correlat* or moderator* or mediator* or determin*)) |
| S21 | S10 OR S11 OR S12 OR S13 OR S14 OR S15 OR S16 OR S17 OR S18 OR S19 OR S20 |
| S20 | (TI(Mixed N1 method*)) or (AB(Mixed N1 method*)) or (KW(Mixed N1 method*)) |
| S19 | (TI (cross sectional N1 (study or studies))) or (AB (cross sectional N1 (study or studies))) or (KW (cross sectional N1 (study or studies))) |
| S18 | (TI(Prospective)) or (AB(Prospective)) or (KW(Prospective)) |
| S17 | (TI(Retrospective)) or (AB(Retrospective)) or (KW(Retrospective)) |
| S16 | (TI(Longitudinal)) or (AB(Longitudinal)) or (KW(Longitudinal)) |
| S15 | (TI (observational N1 (study or studies))) or (AB (observational N1 (study or studies))) or (KW (observational N1 (study or studies))) |
| S14 | (TI (follow up N1 (study or studies))) or (AB (follow up N1 (study or studies))) or (KW (follow up N1 (study or studies))) |
| S13 | (TI (cohort N1 (study or studies))) or (AB (cohort N1 (study or studies))) or (KW (cohort N1 (study or studies))) |
| S12 | (TI (case N1 control)) or (AB (case N1 control)) or (KW (case N1 control)) |
| S11 | (TI (epidemiologic* N1 (study or studies))) or (AB (epidemiologic* N1 (study or studies))) or (KW (epidemiologic* N1 (study or studies))) |
| S10 | questionnaire* or survey* |
| S9 | S4 OR S5 OR S6 OR S7 OR S8 |
| S8 | (TI (quasi N1 (experimental OR randomi*)) or (AB (quasi N1 (experimental OR randomi*))) |
| S7 | (TI (control* N3 (trial* or study or studies)) or (AB (control* N3 (trial* or study or studies)) |
| S6 | (TI (controlled N1 trial*)) or (AB (controlled N1 trial*))) |
| S5 | (TI (randomi* control* trial*)) or (AB (randomi* control* trial*)) |
| S4 | (TI (clinic* N1 (trial* or study or studies))) OR (AB (clinic* N1 (trial* or study or studies))) |
| S3 | S1 OR S2 |
| S2 | acne |
| S1 | DE skin disorders |

Appendix S2 - papers not meeting eligibility criteria excluded at full text

| **Reference** | **Reason for exclusion** | **Study design** |
| --- | --- | --- |
| Abdulrahman N, Alsubeeh, Alsharafi AA, Ahamed SS, Alajlan A. Treatment adherence among patients with five dermatological diseases and four treatment types-a cross-sectional study. Patient Preference and Adherence. 2019;13:2029-38. | Wrong population | Non-RCT |
| Abeck F, Kott J, Bertlich M, Wiesenhutter I, Schroder F, Hansen I, et al. Direct-to-Consumer Teledermatology in Germany: A Retrospective Analysis of 1,999 Teleconsultations Suggests Positive Impact on Patient Care. Telemedicine journal and e-health : the official journal of the American Telemedicine Association. 2023. | Wrong population | Non-RCT |
| Abraham R, Kemeny L, Csoma Z. Patient compliance in acne vulgaris. Journal of the Dermatology Nurses' Association. 2020;12(2). | Wrong publication type (conference abstract) | Non-RCT |
| Acer E, Altinoz AE, Erdogan HK, Agaoglu E, Canakci NY, Saracoglu ZN. Anxiety sensitivity and quality of life in patients with acne. Annals of Dermatology. 2019;31(4):372-7. | Wrong exposure | Non-RCT |
| Afsar FS, Seremet S, Demirlendi Duran H, Karaca S, Mumcu Sonmez N. Sexual quality of life in female patients with acne. Psychology, health & medicine. 2020;25(2):171-8. | Wrong outcomes | Non-RCT |
| Agrawal DA, Khunger N. A Morphological Study of Acne Scarring and Its Relationship between Severity and Treatment of Active Acne. J Cutan Aesthet Surg. 2020;13(3):210-6. | Wrong outcomes | Non-RCT |
| Ahmed F, Hossain A, Begum M, Alam MN, Ibrahim MA. Psychiatric morbidity among the patients with acne Vulgaris in a tertiary care Hospital. Bangladesh Journal of Medical Science. 2019;18(4):773-8. | Wrong exposure | Non-RCT |
| Akinboro AO, Ezejiofor OI, Olanrewaju FO, Oripelaye MM, Olabode OP, Ayodele OE, Onayemi EO. The impact of acne and facial post-inflammatory hyperpigmentation on quality of life and self-esteem of newly admitted nigerian undergraduates. Clinical, Cosmetic and Investigational Dermatology. 2018;11:245-52. | Wrong exposure | Non-RCT |
| Aktepe E, Erturan I, Isik A. Evaluation of problematic Internet usage, characteristics of Internet usage, and other related psychiatric factors in adolescents with acne. Dermatologica Sinica. 2020;38(1):9-14. | Wrong exposure | Non-RCT |
| Al Robaee AA. Assessment of general health and quality of life inpatients with acne using a validated generic questionnaire. Acta Dermatovenerologica Alpina, Pannonica et Adriatica. 2009;18(4):157-64. | Wrong exposure | Non-RCT |
| Alajlan A, Al Turki YA, AlHazzani Y, Alhowaish N, AlEid N, Alhozaimi Z, et al. Prevalence, level of knowledge and lifestyle association with acne vulgaris among medical students. Journal of Dermatology and Dermatologic Surgery. 2017;21(2):58-61. | Wrong population | Non-RCT |
| Alanazi TM, Alajroush W, Alharthi RM, Alshalhoub MZ, Alshehri MA. Prevalence of acne vulgaris, its contributing factors, and treatment satisfaction among the saudi population in Riyadh, Saudi Arabia: A cross-sectional study. Journal of Dermatology and Dermatologic Surgery. 2020;24(1):33-7. | Wrong exposure | Non-RCT |
| Alnahdi, W. A. and Eshmawi, M. and Alharthi, A. and Khafaji, R. and Alhasan, A. and Alqahtani, R. and Alharthi, R. and Kokandi, A. Perception of lifestyle factors and its association with acne vulgaris in Saudi Arabia. British Journal of Dermatology. 2022; 187 (1) | Wrong publication type | Non-RCT |
| Alrabiah, Z. and Wajid, S. and Babelghaith, S. D. and Al Arifi, M. N. Knowledge, attitudes, and practices of community pharmacists toward the management of acne vulgaris in Saudi Arabia. Frontiers in Medicine. 2023; 10 | Wrong outcome | Non-RCT |
| Alruwaili, Maram S. and Alahmadi, Mohammed A. and Alzanadi, Rabab H. and Almughera, Tharaa N. and Almohsen, Danyah S. and Bukhamsin, Sarah A. and Alghamdi, Kholoud M. and Alhaqbani, Renad M. and Alsifri, Sahar S. and AlQahtani, Reemah F. and Alfalah, Haifa A. and Alotaibi, Moteb K. Prevalence of self-prescribed Vitamin A analogs and awareness of related adverse effects. International Journal of Medicine in Developing Countries. 2024; 8 (6) | Wrong outcome | Non-RCT |
| Alshehri MD, Almutairi AT, Alomran AM, Alrashed BA, Kaliyadan F. Over-the-counter and Prescription Medications for Acne: A Cross-Sectional Survey in a Sample of University Students in Saudi Arabia. Indian Dermatol Online J. 2017;8(2):120-3. | Wrong exposure | Non-RCT |
| Al-Shidhani A, Al-Rashdi S, Al-Habsi H, Rizvi S. Impact of acne on quality of life of students at sultan Qaboos University. Oman Medical Journal. 2014;30(1):42-7. | Wrong exposure | Non-RCT |
| Altunisik N, Cansel N, Turkmen D, Ucuz I, Cumurcu HB. Evaluation of temperament and character traits of patients with severe acne. Journal of Cosmetic Dermatology. 2020;19(10):2727 | Wrong exposure | Non-RCT |
| Amr M, Kaliyadan F, Shams T. Use of a cutaneous body image (CBI) scale to evaluate self perception of body image in acne vulgaris. Acta Dermatovenerologica Croatica. 2014;22(3):196-9. | Wrong exposure | Non-RCT |
| Anonymous Clindamycin Plus Benzoyl Peroxide and Adapalene (Cabtreo): Indication: For the topical treatment of acne vulgaris in patients 12 years of age and older: Reimbursement Recommendation. . 2024; | Wrong publication type | Non-RCT |
| Arafa A, Mostafa A, Khamis Y. The association of acne and menstrual symptoms among young women (18-25 years) in Egypt: A population-based cross-sectional study. International Journal of Adolescent Medicine and Health. 2021;33(6):463-8. | Wrong exposure | Non-RCT |
| Bader, Kaitlyn and Andrus, Emma Patients, Providers Seek Therapeutic Alternatives to Benzoyl Peroxide. Dermatology Times. 2024; 45 (7) | Wrong publication type | Non-RCT |
| Bagga, S. and Patel, N. An audit to evaluate the prescribing of antibiotics for acne against NICE guidelines in a digital setting. International Journal of Pharmacy Practice. 2024; 32 | Wrong publication type | Non-RCT |
| Bahaj, R.K. and Alsaggaf, Z. H. and Abduljabbar, M. H. and Hariri, J. O. The Influence of Social media on the Treatment of Acne in Saudi Arabia. Cureus. 2022; 14 (3) | Wrong outcome | Non-RCT |
| Baldwin, H. and Harper, J. C. and Zeichner, J. A. and Draelos, Z. D. and Eichenfield, L. F. and Gold, M. and Gold, L. S. and Kircik, L. Clindamycin Phosphate 1.2%/Adapalene 0.15%/Benzoyl Peroxide 3.1% Gel in Participants With Moderate-to-Severe Acne: The Patient Journey. Journal of Drugs in Dermatology. 2024; 23 (11) | Wrong outcome | Non-RCT |
| Baldwin, H. E. and Graber, E. and Harper, J. C. and Alexis, A. F. and Stein Gold, L. and Kircik, L. and Del Rosso, J. and Hebert, A. A. and Rieder, E. A. and Fried, R. G. and Narayanan, S. and Koscielny, V. and Kasujee, I. INDIVIDUAL ARTICLE: Sarecycline Improves Acne Severity, Symptoms, and Psychosocial Burden in Non-nodular Acne Vulgaris: PROSES Study. Journal of drugs in dermatology : JDD. 2024; 23 (2) | Wrong publication type | Non-RCT |
| Barbieri JS, Hoffstad O, Margolis DJ. Duration of oral tetracycline-class antibiotic therapy and use of topical retinoids for the treatment of acne among general practitioners (GP): A retrospective cohort study. Journal of the American Academy of Dermatology. 2016;75(6):1142-50.e1. | Wrong exposure | Non-RCT |
| Barbieri, J. S. Temporal Trends in the Use of Systemic Medications for Acne From 2017 to 2020. JAMA Dermatology. 2023; 159 (10) | Wrong outcome | Non-RCT |
| Barnes LE, Al-Dabagh A, Huang WW, Feldman SR. Common reasons why acne patients call the office. Dermatology Online Journal. 2014;20(5):3. | Wrong exposure | Non-RCT |
| Barrea L, Donnarumma M, Cacciapuoti S, Muscogiuri G, De Gregorio L, Blasio C, et al. Phase angle and Mediterranean diet in patients with acne: Two easy tools for assessing the clinical severity of disease. Journal of Translational Medicine. 2021;19(1):171. | Wrong exposure | Non-RCT |
| Beisert M, Pilarczyk K, Zakrzewska M, Pawlaczyk M. Sexual satisfaction and self-esteem in women with acne. Journal of Cosmetic Dermatology. 2020;19(7):1768-73. | Wrong exposure | Non-RCT |
| Belmontesi, M. Integrating Dermocosmetic Therapy for Acne: Addressing Severity Levels in Real-Life Experiences. Journal of Drugs in Dermatology. 2025; 24 (1) | Wrong outcome | Non-RCT |
| Bez Y, Yesilova Y, Ari M, Kaya MC, Alpak G, Bulut M. Predictive value of obsessive compulsive symptoms involving the skin on quality of life in patients with acne vulgaris. Acta Dermato-Venereologica. 2013;93(6):679-83. | Wrong exposure | Non-RCT |
| Bhatia K, Mehta V, Bhatia R, Chandnani M, Mehta A. Acne-clinical assessment and impact on quality of life. Australasian Medical Journal. 2012;5(1):44. | Wrong publication type (conference abstract) | Non-RCT |
| Bien, B. and Krajewski, P. and Szepietowski, J. C. Illness acceptance among young adults with acne: a cross-sectional study. Postepy Dermatologii i Alergologii. 2024; 41 (5) | Wrong outcome | Non-RCT |
| Bien, B. and Krajewski, P. K. and Szepietowski, J. C. Acne influences life-changing decisions among young adults. Italian Journal of Dermatology and Venereology. 2024; 159 (2) | Wrong outcome | Non-RCT |
| Bilac O, Bilac C, Tahillioglu A, Uzun AD, Dilcan M, Onder A, et al. The eating attitudes, sleep and personality characteristics, and effects of on acne severity in adolescents with acne vulgaris. Turkish Journal of Dermatology. 2021;15(1):5-10. | Wrong exposure | Non-RCT |
| Bopp, C. and Raab, S. and Yatskayer, M. and Robert, V. and Genesky, G. Clinical evaluation of the effectiveness and consumer perception of an acne spot treatment. Journal of the American Academy of Dermatology. 2017; 76 (6) | Wrong publication type | Non-RCT |
| Bosio Bonet M, Corball MV, Garay IS, Valente E, Ruiz-Lascano A. Epidemiology and impact on quality of life in patients with juvenile acne. Piel. 2023;38(2):83-8. | Wrong exposure | Non-RCT |
| Brajac I, Bilic-Zulle L, Tkalcic M, Loncarek K, Gruber F. Acne vulgaris: Myths and misconceptions among patients and family physicians. Patient Education and Counseling. 2004;54(1):21-5. | Wrong outcomes | Non-RCT |
| Bray JK, Masicampo EJ, Feldman SR. The bandwagon effect increases acne treatment willingness in teenagers. J Am Acad Dermatol. 2020;83(4):1172-4. | Wrong publication type (letter to editor) | Non-RCT |
| Brodell, Robert T. JAAD Game Changers: A retrospective analysis of the duration of oral antibiotic therapy for the treatment of acne among adolescents: investigating practice gaps and potential cost-savings. Journal of the American Academy of Dermatology. 2024; 91 (2) | Wrong publication type | Non-RCT |
| Brook U. Psychosocial impact and personality predictors of coherence feeling among girls with acne. Indian Journal of Pediatrics. 2006;73(3):213-5. | Wrong exposure | Non-RCT |
| Burris J, Rietkerk W, Woolf K. Relationships of self-reported dietary factors and perceived acne severity in a cohort of New York young adults. Journal of the Academy of Nutrition and Dietetics. 2014;114(3):384-92. | Wrong exposure | Non-RCT |
| Carstensen SE, Huang KE, Feldman SR. Treatment failure of patients using topical acne treatments: An observational retrospective cohort study. Journal of Dermatological Treatment. 2014;25(3):193-5. | Wrong exposure | Non-RCT |
| Chatzikonstantinou F, Miskedaki A, Antoniou C, Chatzikonstantinou M, Chrousos G, Darviri C. A novel cognitive stress management technique for acne vulgaris: a short report of a pilot experimental study. Int J Dermatol. 2019;58(2):218-20. | Wrong outcomes | RCT |
| Chilicka K, Rogowska AM, Szygula R, Adamczyk E. Association between satisfaction with life and personality types a and d in young women with acne vulgaris. International Journal of Environmental Research and Public Health. 2020;17(22):1-21. | Wrong outcomes | Non-RCT |
| Choe, J. and Gaurav, A. and Xia, E. and Yan, A. and Mostaghimi, A. and Barbieri, J. S. Use and cost of dietary changes, supplements, over-the-counter topicals, and prescriptions for acne: a cross-sectional survey among United States adults. Archives of Dermatological Research. 2024; 316 (8) | Wrong outcome | Non-RCT |
| Choi JM, Kimball AB. Acne beliefs: Fact and fiction. Cosmetic Dermatology. 2005;18(8):571-7. | Wrong outcomes | Non-RCT |
| Dabash, D. and Salahat, H. and Awawdeh, S. and Hamadani, F. and Khraim, H. and Koni, A. A. and Zyoud, S. H. Prevalence of acne and its impact on quality of life and practices regarding self-treatment among medical students. Scientific reports. 2024; 14 (1) | Wrong outcome | Non-RCT |
| Dalgard F, Gieler U, Holm JO, Bjertness E, Hauser S. Self-esteem and body satisfaction among late adolescents with acne: Results from a population survey. Journal of the American Academy of Dermatology. 2008;59(5):746-51 | Wrong outcomes | Non-RCT |
| Darwish MA, Al-Rubaya AA. Knowledge, beliefs, and psychosocial effect of acne vulgaris among Saudi acne patients. ISRN Dermatology. 2013;2013:929340. | Wrong outcomes | Non-RCT |
| Davis SA, Liu HC, Yu CH, Balkrishnan R, Feldman SR. Underuse of early follow-up visits: A missed opportunity to improve patients' adherence. Journal of Drugs in Dermatology. 2014;13(7):833-6. | Wrong exposure | Non-RCT |
| De Vere Hunt I, Chapman K, Wali G, Bullus S, Fisher R, Matin RN, McPherson T. Establishing and developing a Teenage and Young Adult dermatology clinic with embedded specialist psychological support. Clin Exp Dermatol. 2019;44(8):893-6. | Wrong population | Non-RCT |
| Dessinioti C, Platsidaki E, Zisimou C, Tzanetakou V, Katsambas A, Antoniou C. A cross-sectional study of the perceptions about triggering factors in acne patients. Journal of the European Academy of Dermatology and Venereology. 2018;32(4):e137-e9. | Wrong publication type (letter to editor) | Non-RCT |
| Dhawan, S. S. and Gwazdauskas, J. Clindamycin phosphate 1.2%-benzoyl peroxide (5% or 2.5%) plus tazarotene cream 0.1% for the treatment of acne. Cutis. 2013; 91 (2) | Wrong outcome | Non-RCT |
| Donnarumma M, Fattore D, Ferrillo M, Greco V, Vastarella M, Fabbrocini G. Use of supplementary patient education material increases treatment adherence and quality of life among acne patients in treatment with adapalene 0.3%/benzoyl peroxide 2.5% gel: A clinical study. Journal of the Dermatology Nurses' Association. 2020;12(2). | Wrong publication type (conference abstract) | RCT |
| Draelos Z, Tanghetti E, Guenin E. Vehicle Formulation Impacts Tolerability and Patient Preference: Comparison of Tretinoin Branded Lotion and Generic Cream. Journal of drugs in dermatology : JDD. 2022;21(8):875-80. | Wrong publication type (conference abstract) | RCT |
| Draelos, Z. D. Low irritation potential of tazarotene 0.045% lotion: Head-to-head comparison to adapalene 0.3% gel and trifarotene 0.005% cream in two studies. J Dermatolog Treat. 2023; 34 (1) | Wrong population | Non-RCT |
| Durovic MR, Jankovic J, Durovic M, Spiric J, Jankovic S. Adolescents' beliefs and perceptions of acne vulgaris: A cross-sectional study in Montenegrin schoolchildren. PLoS ONE. 2021;16(6 June):e0253421. | Wrong outcomes | Non-RCT |
| Eastman WJ, Malahias S, Delconte J, DiBenedetti D. Assessing attributes of topical vehicles for the treatment of acne, atopic dermatitis, and plaque psoriasis. Cutis. 2014;94(1):46-53. | Wrong study design | Qualitative |
| Egan N, Loesche MC, Baker MM. Randomized, controlled, bilateral (split-face) comparison trial of the tolerability and patient preference of adapalene gel 0.1% and tretinoin microsphere gel 0.1% for the treatment of acne vulgaris. Cutis; cutaneous medicine for the practitioner. 2001;68(4 Suppl):20-4. | Wrong outcomes | RCT |
| Eichenfield LF, Jarratt M, Schlessinger J, Kempers S, Manna V, Hwa J, et al. Adapalene 0.1% lotion in the treatment of acne vulgaris: Results from two placebo-controlled, multicenter, randomized double-blind, clinical studies. Journal of Drugs in Dermatology. 2010;9(6):639-46 | Wrong outcomes | RCT |
| Eichenfield LF, Nighland M, Rossi AB, Cook-Bolden F, Grimes P, Fried R, Levy S. Phase 4 study to assess tretinoin pump for the treatment of facial acne. Journal of drugs in dermatology : JDD. 2008;7(12):1129-36. | Wrong outcomes | Non-RCT |
| Eichenfield, L. F. and Kwong, P. and Lee, S. and Krowchuk, D. and Arekapudi, K. L. and Hebert, A. A. Advances in Topical Management of Adolescent Facial and Truncal Acne: A Phase 3 Pooled Analysis of Safety and Efficacy of Trifarotene 0.005% Cream. Journal of Drugs in Dermatology. 2022; 21 (6) | Wrong study design | Non-RCT |
| El-Khateeb EA, Khafagy NH, Abd Elaziz KM, Shedid AM. Acne vulgaris: Prevalence, beliefs, patients' attitudes, severity and impact on quality of life in Egypt. Public Health. 2014;128(6):576-8. | Wrong exposure | Non-RCT |
| Ellis RM, Koch LH, McGuire E, Williams JV. Potential barriers to adherence in pediatric dermatology. Pediatric Dermatology. 2011;28(3):242-4 | Wrong population | Non-RCT |
| Erturan İ, Aktepe E, Kocaman O, Sönmez Y, Başak PY, Ceyhan AM, Akkaya VB. Akneli ergenlerin yaşam kalitesi, yalnızlık ve yaşam doyumu düzeylerinin değerlendirilmesi...eti Evaluation of acne quality of life, loneliness and life satisfaction levels in adolescents with acne vulgaris. Archives of the Turkish Dermatology & Venerology / Turkderm. 2014;48(4):172-6. | Wrong exposure | Non-RCT |
| Fabbrocini G, Annunziata MC, Skroza N, Bettoli V, Moftah N, El-Samahy M, et al. Acne and sun exposure: Epidemiology, feelings and behaviours in Mediterranean countries. Journal of Plastic Dermatology. 2013;9(2):101-6. | Wrong exposure | Non-RCT |
| Faisal, U. A. and Jamil, A. and Jaafar, H. and Aqma, W. S. and Arumugam, M. Effects of Malaysian thermal spring water as adjunct therapy for mild to moderate acne vulgaris - a prospective, randomised, controlled, split face study. Medical Journal of Malaysia. 2024; 79 (2) | Wrong outcome | Non-RCT |
| Farid ur R, Niazi NAK. Beliefs and perceptions about acne among undergraduate medical students. Journal of Pakistan Association of Dermatologists. 2007;17(4):231-4. | Wrong population | Non-RCT |
| Feldman W, Hodgson C, Corber S, Quinn A. Health concerns and health-related behaviours of adolescents. Canadian Medical Association Journal. 1986;134(5):489-93 | Wrong population | Non-RCT |
| Felmingham C, Kerr A, Veysey E. Costs incurred by patients with acne prior to dermatological consultation and their relation to patient income. Australas J Dermatol. 2020;61(4):384-6. | Wrong publication type (letter to editor) | Non-RCT |
| Ford CA, Cheek C, Culhane J, Fishman J, Mathew L, Salek EC, et al. Parent and Adolescent Interest in Receiving Adolescent Health Communication Information From Primary Care Clinicians. Journal of Adolescent Health. 2016;59(2):154-61. | Wrong population | Non-RCT |
| Franzke N, Zimmer L, Schafer I, Radermacher C, Kresken J, Augustin M. Quality of medical care of patients with acne vulgaris in Germany - Nationwide survey of pharmacy clients: Original article. JDDG - Journal of the German Society of Dermatology. 2009;7(12):1060-3. | Wrong exposure | Non-RCT |
| Fried R, Nighland M. Acne quality of life and patient satisfaction following treatment with tretinoin pump. Journal of Drugs in Dermatology. 2009;8(12):1080-5. | Wrong exposure | Non-RCT |
| Fried RG, Vamvakias G, Werschler WP. A comparative patient preference study of two gel delivery systems for clindamycin/benzoyl peroxide in the treatment of acne vulgaris. Journal of Adolescent Health. 2009;44(2 SUPPL. 1):S30. | Wrong publication type (conference abstract) | RCT |
| Gokdemir G, Fisek N, Koslu A, Kutlubay Z. Beliefs, perceptions and sociological impact of patients with acne vulgaris in the Turkish population. Journal of Dermatology. 2011;38(5):504-7. | Wrong publication type (letter to editor) | Non-RCT |
| Gold, L. S. and Cruz, A. and Eichenfield, L. and Tan, J. and Jorizzo, J. and Kerrouche, N. and Dhuin, J. C. Effective and safe combination therapy for severe acne vulgaris: a randomized, vehicle-controlled, double-blind study of adapalene 0.1%-benzoyl peroxide 2.5% fixed-dose combination gel with doxycycline hyclate 100 mg. Cutis; cutaneous medicine for the practitioner. 2010; 85 (2) | Wrong outcome | Non-RCT |
| Gold, L. S. and Dashputre, A. A. and Kamleh, M. and Borroto, D. and Lin, T. and Harding, G. and Joseph, G. 40660 Clinically Meaningful Improvement on the Acne Quality of Life Following Treatment with IDP- 126 Gel for Moderate-to-Severe Acne: A Post Hoc Exploratory Pooled Analysis of Phase III Trial Data. Journal of the American Academy of Dermatology. 2023; 89 (3) | Wrong publication type | Non-RCT |
| Gold, L. S. and Dhawan, S. and Weiss, J. and Draelos, Z. D. and Ellman, H. and Stuart, I. Open-label extension study evaluating long-term safety and efficacy of FMX101 4% minocycline foam for moderate-to-severe acne vulgaris. Journal of Clinical and Aesthetic Dermatology. 2019; 12 (10) | Wrong outcome | Non-RCT |
| Gold, L. S. and Harding, G. and Borroto, D. and Choudhury, S. P. and Dutta, S. K. and Lin, T. and Kamleh, M. and Joseph, G. and Dashputre, A. A. Post-Hoc Exploratory Pooled Analysis of the Impact of IDP-126 Gel on QoL in Moderate-to-Severe Acne. Journal of Drugs in Dermatology. 2023; 22 (10) | Wrong outcome | Non-RCT |
| Gollnick HP, Porombka D, Jaeckel A. Adapalene 0.1%/benzoyl peroxide 2.5% in the long-term treatment of moderate to severe acne with or without concomitant medication. Journal of the American Academy of Dermatology. 2015;72(5 SUPPL. 1):AB5 | Wrong publication type (conference abstract) | Non-RCT |
| Gorelick J, Daniels SR, Kawata AK, Degboe A, Wilcox TK, Burk CT, Douse-Dean T. Acne-related quality of life among female adults of different races/ethnicities. Journal of the Dermatology Nurses' Association. 2015;7(3):154-62. | Wrong exposure | Non-RCT |
| Grada A, Perche P, Feldman S. Adherence and Persistence to Acne Medications: A Population-Based Claims Database Analysis. J Drugs Dermatol. 2022;21(7):758-64 | Wrong exposure | Non-RCT |
| Guertler, A. and Neu, K. and Lill, D. and Clanner-Engelshofen, B. and French, L. E. and Reinholz, M. Exploring the potential of omega-3 fatty acids in acne patients: A prospective intervention study. Journal of Cosmetic Dermatology. 2024; 23 (10) | Wrong intervention | Non-RCT |
| Gunduz, B. O. and Atas, H. Relationship between body mass index z-score and acne severity in adolescents: a prospective analysis. Postepy Dermatologii i Alergologii. 2023; 40 (6) | Wrong intervention | Non-RCT |
| Habibuallah, Taha and Malibari, Ward M. and Ghulaysi, Saleh and Tukruni, Omniyyah Abdulsamad and Ahmed, Walaa Abdu and Al-Qozi, Rahaf Ahmed and Almurakshi, Muhjah Mohammed and Sanguf, Mohammed Ahmed and Mojiri, Mohammed Ebrahim Prevalence of acne vulgaris, its contributing factors, and treatment satisfaction among the Saudi population in the Western Region of Saudi Arabia: a cross-sectional study. International Journal of Medicine in Developing Countries. 2024; 8 (7) | Wrong intervention | Non-RCT |
| Hayashi N, Higaki Y, Kawamoto K, Kamo T, Shimizu S, Kawashima M. A cross-sectional analysis of quality of life in Japanese acne patients using the Japanese version of Skindex-16. Journal of Dermatology. 2004;31(12):971-6. | Wrong exposure | Non-RCT |
| Hazarika N, Archana M. The psychosocial impact of acne vulgaris. Indian Journal of Dermatology. 2016;61(5):515-20. | Wrong exposure | Non-RCT |
| Hazarika N, Rajaprabha RK. Assessment of Life Quality Index Among Patients with Acne Vulgaris in a Suburban Population. Indian J Dermatol. 2016;61(2):163-8. | Wrong exposure | Non-RCT |
| Horcajada-Reales C, Conde-Montero E. Juvenile acne: Psychoeducational intervention in 334 adolescents. Piel. 2017;32(8):458-63. | Wrong population | Non-RCT |
| Hudson, Mark P. and Thompson, Andrew R. and Emerson, Lisa-Marie Compassion-focused self-help for psychological distress associated with skin conditions: A randomized feasibility trial. Psychology & Health. 2020; 35 (9) | Wrong population | Non-RCT |
| Huggi, G. and Kamoji, S. and Rayi, M. K. P. and Naveen Kumar, V. and Shabnam, S. Evaluation of Knowledge, Treatment Beliefs and Psychological Impact of Acne Vulgaris in Patients Attending BIMS Hospital: A Cross Sectional Study. International Journal of Pharmaceutical and Clinical Research. 2024; 16 (9) | Wrong outcome | Non-RCT |
| Hung CT, Chen YH, Hung TL, Chiang CP, Chen CY, Wang WM. Clinician-created educational video for shared decision-making in the outpatient management of acne. PLoS ONE. 2022;17(7 July):e0271100. | Wrong population | Non-RCT |
| Huyler AH, Zaenglein AL. Adherence to over-the-counter benzoyl peroxide in patients with acne. Journal of the American Academy of Dermatology. 2017;77(4):763-4. | Wrong publication type (letter to editor) | Non-RCT |
| Hwang EJ, Suh DH. The influence of variable distorted cognition and behavior for the treatment of skin problems. Journal of the American Academy of Dermatology. 2011;64(2 SUPPL. 1):AB40. | Wrong publication type (conference abstract) | Non-RCT |
| Irwin B, Mauriello D, Hemminger L, Pappert A, Kimball AB. Skin sun-acne tutorial evaluation among middle- and high-school students in central New Jersey. Journal of the American Academy of Dermatology. 2007;56(3):407-12. | Wrong population | Non-RCT |
| Israel CE, Ralph-Nwachukwu CI, Attah CA. Knowledge and Treatment Behaviour of Acne Vulgaris among Female Students of University of Nigeria, Enugu Campus, Enugu State, Nigeria. Indian J Dermatol. 2022;67(6):682-7. | Wrong population | Non-RCT |
| Jakubczyk K, Janda K, Chwiłkowska M, Wolska J. [State of knowledge and the prevalence of acne (Acne vulgaris) among young people in Szczecin]. Pomeranian J Life Sci. 2015;61(3):303-9. | Wrong exposure | Non-RCT |
| James Q. A 6% benzoyl peroxide foaming cloth cleanser used in the treatment of acne vulgaris: Aesthetic characteristics, patient preference considerations, and impact on compliance with treatment. Journal of Clinical and Aesthetic Dermatology. 2009;2(7). | Wrong intervention | RCT |
| Jung J, Hwang EJ. Do patients with acne need cognitive behavioral therapy? An analysis of patient knowledge and behavior. International Journal of Dermatology. 2012;51(11):1319-24. | Wrong exposure | Non-RCT |
| Kaikati J, Zoghaib S, Kechichian E, Stephan F, Helou J, Sleilaty G, Tomb R. The impact of acne treatment on quality of life and self-esteem: A prospective cohort study from Lebanon. International Journal of Women's Dermatology. 2021;7(4):415-21. | Wrong exposure | Non-RCT |
| Kane A, Oumou Niang S, Cisse Diagne A, Ly F, Ndiaye B. Epidemiologic, clinical, and therapeutic features of acne in Dakar, Senegal. Nouvelles Dermatologiques. 2007;26(8 SPEC. ISS.):39-41. | Wrong exposure | Non-RCT |
| Kang L, Liu J, An R, Huang J, Huang H, Yi Q. Depression in patients with facial acne vulgaris and the influential factors. Zhong nan da xue xue bao Yi xue ban = Journal of Central South University Medical sciences. 2015;40(10):1115-20 | Wrong exposure | Non-RCT |
| Kansal NK, Hazarika N. The Pattern of Medication Used by Acne Patients: Study from a Tertiary Care Center of North India. Turkish Journal of Dermatology / Turk Dermatoloji Dergisis. 2021;15(2):40-3. | Wrong exposure | Non-RCT |
| Karciauskiene J, Valiukeviciene S, Stang A, Gollnick H. Beliefs, perceptions, and treatment modalities of acne among schoolchildren in Lithuania: A cross-sectional study. International Journal of Dermatology. 2015;54(3):e70-e8. | Wrong exposure | Non-RCT |
| Kaya Ö, Solak SS. Evaluation of the quality, reliability, and popularity of Turkish YouTube videos on acne treatment. Turkderm-Turkish Archives of Dermatology & Venereology. 2022;56(4):166-71. | Wrong exposure | Non-RCT |
| Kaymak Y, Taner E, Taner Y. Comparison of depression, anxiety and life quality in acne vulgaris patients who were treated with either isotretinoin or topical agents. International Journal of Dermatology. 2009;48(1):41-6. | Wrong exposure | Non-RCT |
| Kaymak Y, Ulutas I, Taner E, Bakir B, Simsek I. Body image satisfaction and anxiety of a Turkish sample of university students with skin diseases. Psychological Reports. 2007;100(2):499-508. | Wrong exposure | Non-RCT |
| Kellett SC, Gawkrodger DJ. The psychological and emotional impact of acne and the effect of treatment with isotretinoin. British Journal of Dermatology. 1999;140(2):273-82. | Wrong exposure | Non-RCT |
| Kenyon PE. Psychosomatic aspects of acne: a controlled study. Transactions of the St Johns Hospital Dermatological Society. 1966;52(1):71-8. | Wrong exposure | Non-RCT |
| Khalid, M. and Idrees, M. and Fatima, N. and Jabeen, M. and Wajid, R. Assessment of the frequency of acne and knowledge, attitude and practices for self medication for acne among medical students in Lahore. Journal of Population Therapeutics and Clinical Pharmacology. 2023; 30 (17) | Wrong outcome | Non-RCT |
| Kilkenny M, Merlin K, Plunkett A, Marks R. The prevalence of common skin conditions in Australian school students: 3. Acne vulgaris. British Journal of Dermatology. 1998;139(5):840-5. | Wrong exposure | Non-RCT |
| Kingston, P. and Korouri, E. and Peterson, H. and Lee, K. and Aguero, R. and Huang, M. Y. and Yee, D. and Armstrong, A. W. The effect of perceived health status on satisfaction with care among acne patients: a population-based study. Dermatology Online Journal. 2023; 29 (5) | Wrong publication type | Non-RCT |
| Koch PE, Ryder HF, Dziura J, Njike V, Antaya RJ. Educating adolescents about acne vulgaris: A comparison of written handouts with audiovisual computerized presentations. Archives of Dermatology. 2008;144(2):208-14 | Wrong outcomes | RCT |
| Kosmoski, G. and Du-Soriano, J. and Ilg, D. and DelSasso, A. and Zhang, P. 50551 The Science Behind a Viral Trend: Demonstrating Safety and Efficacy of Hydrocolloid Patch for Facial Acne. Journal of the American Academy of Dermatology. 2024; 91 (3) | Wrong publication type | Non-RCT |
| Kostecka M, Kostecka J, Szwed-Gulaga O, Jackowska I, Kostecka-Jarecka J. The Impact of Common Acne on the Well-Being of Young People Aged 15-35 Years and the Influence of Nutrition Knowledge and Diet on Acne Development. Nutrients. 2022;14(24):5293 | Wrong exposure | Non-RCT |
| Kotekoglu D, Parlakdag A, Koramaz FS, Varol G, Aslankoc V, Bozkurt S, et al. Internalized stigma in acne vulgaris and its relationship with quality of life, general health, body perception, and depression. Nigerian journal of clinical practice. 2020;23(9):1289-94. | Wrong exposure | Non-RCT |
| Krajewska K, Szyszko-Perlowska A, Krajewska-Kulak E, Bartoszewicz A, Heleniak M, Jankowiak B, et al. Knowledge about acne among adolescents from secondary schools in Bialystok. Pediatria Polska. 2004;79(8):631-5. | Wrong population | Non-RCT |
| Krajewska-Kulak E, Krajewska K, Popik M, Heleniak M, Lukaszuk C, Jankowiak B, et al. Application of DLQI index in the assessment of quality of life in adolescents with acne vulagaris. Dermatologia Kliniczna. 2006;8(1):23-6. | Wrong exposure | Non-RCT |
| Kum O, Erturan I, Aktepe E. The evaluation of quality of life, self esteem, peer relationship and anger levels in adolescents with acne at an university hospital: A case control study. Anadolu Psikiyatri Dergisi. 2014;15(2):150-6. | Wrong exposure | Non-RCT |
| Kumar S, Singh R, Kaur S, Mahajan BB. Psychosocial impact of acne on quality of life in North India: A hospital-based cross-sectional study. Journal of Pakistan Association of Dermatologists. 2016;26(1):35-40. | Wrong exposure | Non-RCT |
| Kurhan F, Ozaydin YG, Kubra T. Social appearance anxiety in patients with acne vulgaris: A case-control study. Haseki Tip Bulteni. 2021;59(3):234-8. | Wrong exposure | Non-RCT |
| Ladbrooke S, Waterman M, El-Naes R, Finch C, Fryatt E, Allgar V, et al. A pilot study comparing the impact of acne on the quality of life of women with persistent acne vs. Late-onset acne. British Journal of Dermatology. 2014;171(SUPPL. 1):44. | Wrong publication type (conference abstract) | Non-RCT |
| Langenbruch AK, Schafer I, Franzke N, Augustin M. Participants of online surveys on health care in dermatology have something incommon: The critical view. Experimental Dermatology. 2010;19(2):222. | Wrong publication type (conference abstract) | Non-RCT |
| Lee SH, Cho HS, Seung NR, Jung SJ, Kim CW, Jo HJ, et al. Quality of life of acne patients. Korean Journal of Dermatology. 2006;44(6):688-95. | Wrong exposure | Non-RCT |
| Li, M. and Lynde, C. and Sibley, C. and Bernstein, S. C. and Mathieu, S. and Guenther, L. and Dayeh, N. R. and Tan, J. A Multicentre Evaluation of a Ceramide-Containing Hydrating Cream-to-Foam Cleanser and Facial Moisturizing Lotion for Improving Topical Treatment Tolerability in Facial Acne. Journal of Cutaneous Medicine and Surgery. 2025; 29 (1) | Wrong outcome | Non-RCT |
| Liu YS, Lu NH, Shieh PC, Sun CK. Combination of a Self-Regulation Module and Mobile Application to Enhance Treatment Outcome for Patients with Acne. Medicina (Kaunas, Lithuania). 2020;56(6). | Wrong exposure | Non-RCT |
| Loney T, Standage M, Lewis S. Not just 'skin deep': Psychosocial effects of dermatological-related social anxiety in a sample of acne patients. Journal of Health Psychology. 2008;13(1):47-54. | Wrong exposure | Non-RCT |
| Maiprasert, M. and Khunngam, P. and Rakchart, S. and Tovanabutr, N. and Wanitphakdeedecha, R. and Srinoulprasert, Y. and Chai-Adisaksopha, C. Elimination Diet Guided by Food-Specific IgG Antibodies Measurements in Chronic Adult Acne in Thailand: A Prospective RCT Study. International Journal of Statistics in Medical Research. 2024; 13 | Wrong intervention | Non-RCT |
| Maisonneuve H, Cambazard F, Levy E, Thivolet J. Evaluation of the number and cost of severe acne in France. Annales de Dermatologie et de Venereologie. 1987;114(10):1203-9. | Wrong exposure | Non-RCT |
| Malave, G. S. and Fitzsimmons, R. and Shin, D. B. and Takeshita, J. 687 Acne-related quality of life differs by race/ethnicity and sex. Journal of Investigative Dermatology. 2022; 142 (8) | Wrong study design | Non-RCT |
| Markovic M, Soldatovic I, Bjekic M, Sipetic-Grujicic S. Adolescents' self perceived acne-related beliefs: from myth to science. An Bras Dermatol. 2019;94(6):684-90. | Wrong exposure | Non-RCT |
| Martel J, Powell E, Murina A. The effect of Instagram and photograph editing on seeking dermatologic care. Journal of Cosmetic Dermatology. 2020;19(10):2732-5. | Wrong exposure | Non-RCT |
| McLellan C, O'Neil AI, Cameron M, Reed A, Henderin C, Dellavalle RP, Tan J. Facilitating Informed Treatment Decisions in Acne: A Pilot Study of a Patient Decision Aid. Journal of Cutaneous Medicine and Surgery. 2019;23(1):117-8. | Wrong population | Non-RCT |
| Mills OH, Berger RS, Kligman AM, McElroy JA, Di Matteo J. A comparative study of Erycette vs Cleocin-T. Advances in Therapy. 1992;9(1):14-20. | Wrong outcomes | RCT |
| Min, M. and Malhi, J. K. and Chambers, C. J. and Sivamani, R. Impact of Pediatric Dermatologic Conditions on Child and Parent Quality of Life. Cureus. 2023; 15 (7) | Wrong population | Non-RCT |
| Misery L, Wolkenstein P, Amici JM, Maghia R, Brenaut E, Cazeau C, et al. Consequences of acne on stress, fatigue, sleep disorders and sexual activity: A population-based study. Acta Dermato-Venereologica. 2015;95(4):485-8. | Wrong outcomes | Non-RCT |
| Mohammed Lotfy, A. M. and Anwar, M. M. and Nadi, A. B. and Ahmed, G. S. Knowledge, attitudes, and practices regarding self-medication for acne among medical students in Beni-Suef University. International Journal of Adolescent Medicine and Health. 2024; 36 (4) | Wrong outcome | Non-RCT |
| Moosa AS, Lim SF, Koh YLE, Aau WK, Tan NC. The management of acne vulgaris in young people in primary care: A retrospective cohort study. Frontiers in Medicine. 2023;10:1152391. | Wrong outcomes | Non-RCT |
| Mosam A, Vawda NB, Gordhan AH, Nkwanyana N, Aboobaker J. Quality of life issues for South Africans with acne vulgaris. Clinical and Experimental Dermatology. 2005;30(1):6-9. | Wrong outcomes | Non-RCT |
| Mulder MMS, Sigurdsson V, Van Zuuren EJ, Klaassen EJ, Faber JAJ, De Wit JBF, Van Vloten WA. Psychosocial impact of acne vulgaris: Evaluation of the relation between a change in clinical acne severity and psychosocial state. Dermatology. 2001;203(2):124-30. | Wrong outcomes | Non-RCT |
| Niemeier V, Kupfer J, Demmelbauer-Ebner M, Slangier U, Effendy I, Gieler U. Coping with acne vulgaris. Evaluation of the chronic skin disorder questionnaire in patients with acne. Dermatology. 1998;196(1):108-15. | Wrong exposure | Non-RCT |
| Okhovat JP, Tenconi F, Kim J, Kim CN. The Utility of Text Message Reminders for Acne Patients: A Pilot Study. American Journal of Clinical Dermatology. 2017;18(1):133-7. | Wrong population | Non-RCT |
| Ozkesici Kurt B. Comparison of the psychosocial impact of acne in adolescents and adults; body satisfaction, self-esteem, and quality of life. Journal of Cosmetic Dermatology. 2022;21(2):836-43. | Wrong outcomes | Non-RCT |
| Ozolins M, Eady EA, Avery A, Cunliffe WJ, O'Neill C, Simpson NB, Williams HC. Randomised controlled multiple treatment comparison to provide a cost-effectiveness rationale for the selection of antimicrobial therapy in acne. Health technology assessment (Winchester, England). 2005;9(1):iii-212. | Wrong outcomes | RCT |
| Öztekin C, Öztekin A. The association of depression, loneliness and internet addiction levels in patients with acne vulgaris. Biopsychosoc Med. 2020;14:17. | Wrong outcomes | Non-RCT |
| Ozturk P, Orhan FO, Dokur N, Ozer A. Assesment of temperament and character profile of patients with acne vulgaris. Noropsikiyatri Arsivi. 2012;49(4):255-9 | Wrong exposure | Non-RCT |
| Ozturk P, Ozlem Orhan F, Ozer A, Karakas T, Nuri Oksuz A, Yalcin Yetisir N. Assessment of temperament and character profile with anxiety and depression in patients with acne. Balkan Medical Journal. 2013;30(2):161-6. | Wrong outcomes | Non-RCT |
| Ozyay Eroglu F, Aktepe E, Erturan I. The evaluation of psychiatric comorbidity, self-injurious behavior, suicide probability, and other associated psychiatric factors (lonileness, self-esteem, life satisfaction) in adolescents with acne: A clinical pilot study. Journal of Cosmetic Dermatology. 2019;18(3):916-21. | Wrong exposure | Non-RCT |
| Perveen, Z. and Zulfiqar, M. and Raza, A. and Umbreen, S. and Liaqat, Z. and Saeed, M. and Sheikh, N. H. and Naqvi, S. Z. H. Knowledge and Practices towards Acne Vulgaris among Acne patients in a tertiary care hospital, Dina Nath, Pakistan. Pakistan Journal of Medical and Health Sciences. 2023; 17 (3) | Wrong outcome | Non-RCT |
| Piccardi, N. and Chan, K. and Grivet-Seyve, M. and Lachmann, N. and Chavda, R. 51725 Importance of a tailored skin care routine for patients with moderate acne vulgaris and post-inflammatory hyperpigmentation treated with topical trifarotene. Journal of the American Academy of Dermatology. 2024; 91 (3) | Wrong publication type | Non-RCT |
| Pinto, A. L. and Ferreira, C. and Teixeira, M. L. and Rosa, M. and Paulino, E. Living the skin: A community pharmacy-based follow-up programme for acne, atopic dermatitis and hyperpigmentation. Pharmacy Education. 2023; 23 (3) | Wrong publication type | Non-RCT |
| Pollack A, Preda V, Sullivan J. Future practitioners; Medical and pharmacy student knowledge of acne. Australasian Journal of Dermatology. 2009;50(SUPPL. 1):A37. | Wrong publication type (conference abstract) | Non-RCT |
| Purvis D, Robinson E, Merry S, Watson P. Acne, anxiety, depression and suicide in teenagers: a cross-sectional survey of New Zealand secondary school students. Journal of Paediatrics & Child Health. 2006;42(12):793-6. | Wrong exposure | Non-RCT |
| Qi He Leow M, Taige CAO. Perception on skin problems, skin care and knowledge needs of people in the Singapore community. Singapore Nursing Journal. 2018;45(1):20-6. | Wrong population | Non-RCT |
| Rashid, S. and Jamil, Z. and Azeem, S. and Afzal, N. Living with acne: A patient-centered study on myths, beliefs and perceptions. Journal of Pakistan Association of Dermatologists. 2023; 33 (4) | Wrong outcome | Non-RCT |
| Rasool, F. and Khan, H. M. and Waheed, A. and Akhtar, N. Acne vulgaris: Prevalence, clinical forms and its management in pharmacy students from Bahawalpur, Pakistan. Value in Health. 2017; 20 (9) | Wrong publication type | Non-RCT |
| Richard, M. A. and Paul, C. and Nijsten, T. and Gisondi, P. and Salavastru, C. and Taieb, C. and Stratigos, A. and Trakatelli, M. and Puig, L. The journey of patients with skin diseases from the first consultation to the diagnosis in a representative sample of the European general population from the EADV burden of skin diseases study. Journal of the European Academy of Dermatology and Venereology. 2023; 37 | Wrong population | Non-RCT |
| Rieder, E. A. and Andriessen, A. and Cutler, V. and Gonzalez, M. E. and Greenberg, J. L. and Lio, P. and Love, E. M. and Oza, V. and Park, J. H. and Andriessen, H. and Phillips, K. A. Dermatology in Contemporary Times: Building Awareness of Social Media's Association With Adolescent Skin Disease and Mental Health. Journal of Drugs in Dermatology. 2023; 22 (8) | Wrong study design | Non-RCT |
| Ritvo E, Del Rosso JQ, Stillman MA, La Riche C. Psychosocial judgements and perceptions of adolescents with acne vulgaris: A blinded, controlled comparison of adult and peer evaluations. Biopsychosoc Med. 2011;5(1):11. | Wrong outcomes | Non-RCT |
| Rodriguez D, Davis MW. The BEST study: results according to prior treatment. Cutis; cutaneous medicine for the practitioner. 2003;71(2 Suppl):27-34. | Wrong outcomes | Non-RCT |
| Ruggiero G, Arcangeli F, Feliciangeli M, Sacchetti R, Calzaretti R, Bonfanti R, et al. Acne and primary care paediatrician. Medico e Bambino. 2012;31(7):449-53. | Wrong exposure | Non-RCT |
| Ryskina KL, Goldberg E, Lott B, Hermann D, Barbieri JS, Lipoff JB. The role of the physician in patient perceptions of barriers to primary adherence with acne medications. JAMA Dermatology. 2018;154(4):456-9. | Wrong study design | Qualitative |
| Sablosky L. Teenagers with acne: a survey of their attitudes towards skin problems and treatment by the physician. Am Pract Dig Treat. 1961;12:515-6 | Wrong outcomes | Non-RCT |
| Sachar M, Xiong M, Lee KC. Consumer preferences of top-rated over-the-counter acne treatment products: a cohort study. Archives of Dermatological Research. 2022;314(8):815-21 | Wrong exposure | Non-RCT |
| Seite S, Caixeta C, Towersey L. Large-scale survey to describe acne management in Brazilian clinical practice. Clinical, Cosmetic and Investigational Dermatology. 2015;8:571-7. | Wrong exposure | Non-RCT |
| Seite S, Rougier A, Dreno B. An investigational study of acne treatment in France. Annales de Dermatologie et de Venereologie. 2012;139(10):611-6. | Wrong exposure | Non-RCT |
| Sereflican B, Tuman TC, Tuman BA, Parlak AH. Type D personality, anxiety sensitivity, social anxiety, and disability in patients with acne: A cross-sectional controlled study. Postepy Dermatologii i Alergologii. 2019;36(1):51-7 | Wrong exposure | Non-RCT |
| Shafi Z, Ullah MR, Qayyum S, Waqar HY. Do our students know about acne: A cross sectional study from King Edward Medical University, Lahore. Pakistan Journal of Medical and Health Sciences. 2018;12(3):1230-2 | Wrong outcomes | Non-RCT |
| Shaukat S, Aman S, Hussain I, Kazmi AH. The effect of oral doxycycline and topical 5% benzoyl peroxide on quality of life in patients with mild to moderate acne vulgaris. Journal of Pakistan Association of Dermatologists. 2013;23(2):173-9. | Wrong exposure | Non-RCT |
| Shields, A. and Nock, M. R. and Ly, S. and Manjaly, P. and Mostaghimi, A. and Barbieri, J. S. Evaluation of Stigma Toward Individuals with Acne. JAMA Dermatology. 2024; 160 (1) | Wrong population | Non-RCT |
| Singam V, Rastogi S, Patel KR, Lee HH, Silverberg JI. The mental health burden in acne vulgaris and rosacea: an analysis of the US National Inpatient Sample. Clinical and Experimental Dermatology. 2019;44(7):766-72. | Wrong population | Non-RCT |
| Singh IP, Poonia K, Bajaj K. Quality of life in young adults with acne: Results of a cross-sectional study. Journal of Cosmetic Dermatology. 2021;20(12):4017-23. | Wrong exposure | Non-RCT |
| Sorrell J, Schlosser BJ, Bayers S, Becker L, Rademaker A, Chamlin SL. Acne vulgaris and quality of life: A comparison of teens and their parents. Pediatric Dermatology. 2013;30(5):653. | Wrong publication type (conference abstract) | Non-RCT |
| Stewart TJ, Bazergy C. Hormonal and dietary factors in acne vulgaris versus controls. Dermato-Endocrinology. 2018;10(1):e1442160. | Wrong exposure | Non-RCT |
| Straight CE, Lee YH, Liu G, Kirby JS. Duration of oral antibiotic therapy for the treatment of adult acne: A retrospective analysis investigating adherence to guideline recommendations and opportunities for cost-savings. Journal of the American Academy of Dermatology. 2015;72(5):822-7. | Wrong exposure | Non-RCT |
| Suh DH, Oh H, Lee SJ, Kim HJ, Ryu HJ. Relationship between acne and the use of cosmetics: Results of a questionnaire study in 539 Korean individuals. Journal of Cosmetic Dermatology. 2021;20(7):2172-8. | Wrong exposure | Non-RCT |
| Sultan, N. and Iftikhar, A. and Hashmi, S. and Rehman, S. and Butt, G. and Rana, M. Why medical students don't seek treatment for acne vulgaris, patient's perspective. Journal of Pakistan Association of Dermatologists. 2023; 33 (4) | Wrong outcome | Non-RCT |
| Swink SM, Jones-Sheets MA, Sinharoy A, Butt M, Gust A, Zaenglein AL. Evaluating Dermatology Residents Combined Oral Contraceptive Pill Prescribing Practices, Knowledge, and Comfort Level in the Management of Acne Vulgaris: A Cross-sectional Survey Study. Journal of Clinical and Aesthetic Dermatology. 2023;16(1):47-50. | Wrong exposure | Non-RCT |
| SzabÃ³, Ã and Brodszky, V. and Rencz, F. Comparing EQ-5D-5L, PROPr, SF-6D and TTO utilities in patients with chronic skin diseases. Eur J Health Econ. 2024; | Wrong population | Non-RCT |
| Szepietowska, M. and Bien, B. and Krajewski, P. K. and Stefaniak, A. A. and Matusiak, L. Prevalence, Intensity and Psychosocial Burden of Acne Itch: Two Different Cohorts Study. Journal of Clinical Medicine. 2023; 12 (12) | Wrong outcome | Non-RCT |
| Szepietowski JC, Wolkenstein P, Veraldi S, Tennstedt D, Machovcova A, Delarue A. Acne across Europe: an online survey on perceptions and management of acne. Journal of the European Academy of Dermatology and Venereology. 2018;32(3):463-6. | Wrong outcomes | Non-RCT |
| SzlÃ¡vicz, Eszter and SzabÃ³, Ãkos and KinyÃ³, Ãgnes and Szeiffert, Anita and BancsÃ³k, TamÃ¡s and Brodszky, Valentin and Gyulai, Rolland and Rencz, Fanni Content validity of the EQ-5D-5L with skin irritation and self-confidence bolt-ons in patients with atopic dermatitis: A qualitative think-aloud study. Quality of Life Research: An International Journal of Quality of Life Aspects of Treatment, Care & Rehabilitation. 2024; 33 (1) | Wrong population | Non-RCT |
| Taalsiewicz K, Odlakowska A, Szczerkowska-Dobosz A. Evaluation of knowledge about acne vulgaris among a selected population of adolescents of Tricity schools. Postepy Dermatologii i Alergologii. 2012;29(6):417-20. | Wrong population | Non-RCT |
| Tabatabaei F, Pasalar M, Tajadini H, Kamali M, Rampp T. Effectiveness of Wet Cupping on Patients with Facial Acne Vulgaris: A 12-Week, Randomized, Single-Blind, Intervention-Sham-Controlled Trial. Complementary Medicine Research. 2021;28(6):508-15. | Wrong exposure | Non-RCT |
| Tadinac-Babic M, Kotrulja L, Oremovic L, Poduje S. Quality of life in patients with acne. Socijalna Psihijatrija. 2001;29(4):194-8. | Wrong exposure | Non-RCT |
| Taha, S. and Shakhshir, M. and Zyoud, S. H. Acne Vulgaris and adherence to the mediterranean diet among university students: a case-control study. Journal of health, population, and nutrition. 2024; 43 (1) | Wrong outcome | Non-RCT |
| Tallab TM. Beliefs, perceptions and psychological impact of acne vulgaris among patients in the Assir region of Saudi Arabia. West African Journal of Medicine. 2004;23(1):85-7. | Wrong outcomes | Non-RCT |
| Tan HH, Tan AW, Barkham T, Yan XY, Zhu M. Community-based study of acne vulgaris in adolescents in Singapore. Br J Dermatol. 2007;157(3):547-51. | Wrong outcomes | Non-RCT |
| Tan JKL, Vasey K, Fung KY. Attitudes of female patients regarding oral contraceptives for treatment of acne. Journal of Cutaneous Medicine and Surgery. 2001;5(6):471-4. | Wrong outcomes | Non-RCT |
| Tan JKL, Vasey K, Fung KY. Beliefs and perceptions of patients with acne. Journal of the American Academy of Dermatology. 2001;44(3):439-45. | Wrong outcomes | Non-RCT |
| Tang J, Yang J, Zhang C, Zhang X, Chen Q, Zhang S, et al. The factors influencing the unmet effects of alpha hydroxy acid peels on acne vulgaris: A single tertiary center experience. Dermatologic Therapy. 2021;34(4):e14956. | Wrong exposure | Non-RCT |
| Temel AB, Bozkurt S, Senol Y, Alpsoy E. Internalized stigma in patients with Acne Vulgaris, Vitiligo, and alopecia areata. Turkish Journal of Dermatology. 2019;13(3):109-16. | Wrong exposure | Non-RCT |
| Ting, W. Randomized, observer-blind, split-face study to compare the irritation potential of 2 topical acne formulations over a 14-day treatment period. Cutis; cutaneous medicine for the practitioner. 2012; 90 (2) | Wrong outcome | Non-RCT |
| Toy J, Wan V, Lee DG, Liu C, Fleming P, Lynde C. Perspectives and knowledge of acne vulgaris among young adolescents. Pediatric Dermatology. 2023;40(2):308-11. | Wrong population | Non-RCT |
| Tran, H. W. and Le, P. Impact of clinical pharmacist-led isotretinoin management and acne care education at a multispecialty clinic. British Journal of Dermatology. 2023; 188 | Wrong publication type | Non-RCT |
| Tsoy N. The influence of dietary habits on acne. World Journal of Medical Sciences. 2013;8(3):212-6 | Wrong exposure | Non-RCT |
| Tucker R, MacLure K, Paudyal V, Stewart D. Exploring the diagnosis and management of dermatitis and acne by community pharmacists. International Journal of Pharmacy Practice. 2017;25(Supplement 1):17. | Wrong publication type (conference abstract) | Non-RCT |
| Tucker RP. An exploration of the range of skin problems encountered in community pharmacy and the extent to which pharmacists undertake dermatology medicine use reviews. International Journal of Pharmacy Practice. 2012;20(SUPPL. 1):44-5. | Wrong publication type (conference abstract) | Non-RCT |
| Tuong W, Wang AS, Armstrong AW. Effect of Automated Online Counseling on Clinical Outcomes and Quality of Life Among Adolescents With Acne Vulgaris: A Randomized Clinical Trial. JAMA Dermatol. 2015;151(9):970-5. | Wrong outcomes | RCT |
| Uhlenhake E, Yentzer BA, Feldman SR. Acne vulgaris and depression: A retrospective examination. Journal of Cosmetic Dermatology. 2010;9(1):59-63. | Wrong exposure | Non-RCT |
| Ukonu AB, Ibekwe PE, Ezechukwu A. Anxiety state and its psychosocial consequences among acne vulgaris and chronic urticaria patients in Abuja. International Journal of Psychological Studies. 2016;8(1):1-10. | Wrong exposure | Non-RCT |
| Unlu Acikel S, Aktas A, Caykoylu A, Kalkan G. Comparison of acne vulgaris patients and healthy controls in terms of autistic features and 2D:4D ratio. International Journal of Developmental Disabilities. 2023. | Wrong exposure | Non-RCT |
| Vanoosthuyze K, Layton AM. Knowledge, perceptions and impacts of puberty on the hair and skin in males: A global survey. Journal of the Dermatology Nurses' Association. 2020;12(2). | Wrong publication type (conference abstract) | Non-RCT |
| Vishakha, Geetha A. Cost Variation Analysis of various brands of topical medications used in Acne vulgaris currently available in Indian Pharmaceutical Market. Indian Journal of Physiology and Pharmacology. 2018;62(Supplement 1):198-9. | Wrong population | Non-RCT |
| Walker N, Lewis-Jones MS. Quality of life and acne in Scottish adolescent schoolchildren: use of the Children's Dermatology Life Quality Index (CDLQI) and the Cardiff Acne Disability Index (CADI). J Eur Acad Dermatol Venereol. 2006;20(1):45-50. | Wrong exposure | Non-RCT |
| Wan, V. and Selvakumar, R. and Toy, J. and Lee, D. G. and Zhang, Q. and Fleming, P. 44596 The Acne Education Project: An Educational Initiative to Improve Acne Health Literacy and Increase Help-seeking Behaviour in Pre-pubescent Youth. Journal of the American Academy of Dermatology. 2023; 89 (3) | Wrong population | Non-RCT |
| Wan, V. and Selvakumar, R. and Zhang, Q. and Fleming, P. and Lynde, C. The Acne Education Project: An educational initiative to improve acne health literacy and promote help-seeking behavior in young adolescents. Pediatric Dermatology. 2024; 41 (1) | Wrong population | Non-RCT |
| Wang A, Armstrong A, Schupp C, Wu J. Randomized controlled trial examining effectiveness of a novel personalized interactive health care education tool for acne patients (MyPACE). Journal of the American Academy of Dermatology. 2013;68(4 SUPPL. 1):AB18. | Wrong publication type (conference abstract) | RCT |
| Warburton K, Whitehouse H, El-Naes R, Eady A, Layton A. How people with acne decide whether their treatment is working. British Journal of Dermatology. 2014;171(SUPPL. 1):38. | Wrong publication type (conference abstract) | Non-RCT |
| Wen L, Jiang G, Zhang X, Lai R, Wen X. Relationship between acne and psychological burden evaluated by ASLEC and HADS surveys in high school and college students from central China. Cell biochemistry and biophysics. 2015;71(2):1083-8. | Wrong exposure | Non-RCT |
| Whitehouse HJ, Eady EA, Ward CJ, Layton AM. Prescribing for acne in the U.K.: Patterns and influencers. British Journal of Dermatology. 2016;175(Supplement 1):34. | Wrong publication type (conference abstract) | Non-RCT |
| Wu TQ, Mei SQ, Zhang JX, Gong LF, Wu FJ, Wu WH, et al. Prevalence and risk factors of facial acne vulgaris among Chinese adolescents. International Journal of Adolescent Medicine and Health. 2007;19(4):407-12. | Wrong exposure | Non-RCT |
| Yang, F. and Wang, H. and Guo, M. and Zhou, Z. The clinical efficacy of a new emulsion for acne and conspicuous facial pore amelioration. Journal of Cosmetic Dermatology. 2024; 23 (3) | Wrong outcome | Non-RCT |
| Yap FBB. Cost of medications in the treatment of acne in east malaysian borneo. Journal of the Dermatology Nurses' Association. 2020;12(2). | Wrong publication type (conference abstract) | Non-RCT |
| Yarpuz AY, Saadet ED, Şanli HE, Özgüven HD. Akne vulgaris hastalarinda sosyal kaygi düzeyi ve buhun klinik değişkenler ile ilişkisi = Social anxiety level in acne vulgaris patients and its relationship to clinical variables. Türk Psikiyatri Dergisi. 2008;19(1):29-37 | Wrong exposure | Non-RCT |
| Yoqub F, Bajwa RS, Batool I. Impact of acne vulgaris on self-esteem, body image perception and quality of life among adolescents. Journal of Pakistan Association of Dermatologists. 2020;30(1):26-31. | Wrong exposure | Non-RCT |
| Yu, Z. and Swenson, K. and Graber, E. Prospective Pilot Evaluation of the Safety, Tolerability, and Efficacy of Clindamycin Phosphate 1.2%/Adapalene 0.15%/Benzoyl Peroxide 3.1% Gel plus Clascoterone 1% Cream in Adult Patients with Acne. Journal of Clinical and Aesthetic Dermatology. 2025; 18 (1) | Wrong outcome | Non-RCT |
| Zhang Y, Wu H, Tu C, Sun Y, Xiao B, Guo H, et al. The assessment of psychology, quality of life in acne patients and evaluation of ALA-PDT for moderate to severe acne. Photodiagnosis and Photodynamic Therapy. 2021;33:102099. | Wrong exposure | Non-RCT |
| Zhang, Z. and Zhou, M. The impact of social media information exposure on appearance anxiety in young acne patients: a moderated chain mediation model. Front Psychol. 2024; 15 | Wrong outcome | Non-RCT |
| Zhu J, Peng K, Zhang Y, Bai X, Zhong C, Ye J, Lu M. Sleep quality, circadian preferences, and mood among patients with acne vulgaris: a case-control study. Sleep and Breathing. 2023. | Wrong exposure | Non-RCT |
| Zujko-Kowalska, K. and Jankowska, B. and Zujko, M. E. The Antioxidant Power of a Diet May Improve the Quality of Life of Young Women with Acne Vulgaris. Nutrients. 2024; 16 (9) | Wrong outcome | Non-RCT |

Appendix S3 - inclusion and exclusion criteria for RCTs

Population

We included RCTs of people of all ages with acne vulgaris, provided some were aged between 13-25 (their data did not need to be reported separately), health practitioners treating acne and carers/parents of children with acne vulgaris. We included RCTS concerning multiple skin conditions if data for acne alone in the aforementioned population were reported separately.

Intervention and comparators

Interventions eligible for inclusion were medicinal and/or behavioural or complex interventions that offered a combination of treatment and management approaches and included some element of quantitative process evaluation (e.g., investigating factors associated with engagement with, adherence to, or outcomes of the intervention). We excluded studies were interventions concerned only oral-isotretinoin/Roaccutane, the mental health impact of acne or acne scarring or post-inflammatory hyperpigmentation (as opposed to active acne treatment). Any comparator was eligible for inclusion.

Outcomes

Of interest were quantitative data for predictors of/correlates of/factors associated with, and/or relationships between adherence and/or engagement with the intervention, and any of the following outcomes:

•	acne symptom severity

•	acne-related quality of life as measured by patient reported outcome measures e.g. ACNE-QoL, CompAQ, ACNE-Q etc.

•	adherence/compliance to acne treatments

•	acne help-seeking behaviour in relation to acne treatment and management

•	knowledge and /or beliefs about acne

•	healthcare care professionals’ acne treatment/management related decisions or behaviours

•	Out-of-pocket costs incurred by young people and their families

Language and Publication types

There were no language restrictions. Non-English language articles were translated to English. We excluded RCTs reported in conference abstracts only as scoping revealed these were unlikely to contain the quantitative outcome related data of interest.

Appendix S4 - papers included in systematic of RCTs of interventions for adherence to acne treatments

| **Reference** | **Reason for exclusion** | **Study design** |
| --- | --- | --- |
| Boker, A., et al. (2012). Do automated text messages increase adherence to acne therapy? Results of a randomized, controlled trial. Journal of the American Academy of Dermatology 67(6): 1136-1142. | Reported separately | RCT |
| Donnarumma, M., et al. (2019). How to Increase Adherence and Compliance in Acne Treatment? A Combined Strategy of SMS and Visual Instruction Leaflet. Dermatology 235(6): 463-470. | Reported separately | RCT |
| Fabbrocini, G., et al. (2014). Acne Smart Club: An educational program for patients with Acne. Dermatology 229(2): 136-140. | Reported separately | RCT |
| Fabbrocini, G., et al. (2016). Informative flashcards to increase therapy compliance in acne patients receiving adapalene 0.1%/benzoyl peroxide 2.5. Esperienze Dermatologiche 8(3-4): 178-182. | Reported separately | RCT |
| Hayashi, N. and M. Kawashima (2014). Study of the usefulness of moisturizers on adherence of acne patients treated with adapalene. Journal of Dermatology 41(7): 592-597. | Reported separately | RCT |
| Myhill, T., et al. (2017). Use of Supplementary Patient Education Material Increases Treatment Adherence and Satisfaction Among Acne Patients Receiving Adapalene 0.1%/Benzoyl Peroxide 2.5% Gel in Primary Care Clinics: A Multicenter, Randomized, Controlled Clinical Study. Dermatology and Therapy 7(4): 515-524. | Reported separately | RCT |
| Navarrete-Dechent, C., et al. (2015). Oral and written counseling is a useful instrument to improve short-term adherence to treatment in acne patients: a randomized controlled trial. Dermatol Pract Concept 5(4): 13-16. | Reported separately | RCT |
| Sandoval, L. F., et al. (2014). Pilot randomized-control trial to assess the effect product sampling has on adherence using adapalene/benzoyl peroxide gel in acne patients. Journal of Drugs in Dermatology 13(2): 135-140. | Reported separately | RCT |
| Yentzer, B. A., et al. (2010). Simplifying regimens promotes greater adherence and outcomes with topical acne medications: a randomized controlled trial. Cutis 86(2): 103-108. | Reported separately | RCT |

Appendix S5 - Papers met original eligibility criteria but did not include data on adherence to specific acne treatments

| **Reference** | **Study design** |
| --- | --- |
| Ablon, G. A Seven-week, Open-label Trial Evaluating the Safety and Efficacy of a Photopneumatic Device for Mitigating Mild-to-Moderate Acne in Healthy Adolescents and Young Adults. Journal of Clinical and Aesthetic Dermatology. 2024; 17 (6) | Non-RCT |
| Achavanuntakul P, Oulansakoonchai N, Phanpanich W, Wongsena P, Phanpanich P, Indra T, et al. Questionnaire study of preferences in acne treatments among first-year students in ubon ratchathani university. Journal of the Medical Association of Thailand. 2018;101(10):1409-15. | Non-RCT |
| Ahmad A, Alghanemi L, Alrefaie S, Alorabi S, Ahmad G, Zimmo S. The use of complementary medicine among acne valguris patients: Cross sectional study. Journal of Dermatology and Dermatologic Surgery. 2017;21(2):66-71. | Non-RCT |
| Al Robaee AA. Prevalence, knowledge, beliefs and psychosocial impact of acne in University students in Central Saudi Arabia. Saudi Medical Journal. 2005;26(12):1958-61. | Non-RCT |
| Alghofaili, A., et al. (2021). The effect of depression on treatment adherence among a sample of saudi patients diagnosed with acne vulgaris. Clinical, Cosmetic and Investigational Dermatology 14: 1497-1506. | Non-RCT |
| Alkhawaja E, Alkhawaja B, Hamadi S, Abdelmalek M, Abdelmalek SMA. Perceptions, beliefs, knowledge, and psychological impact of acne vulgaris in jordan: Acne from the community perspective. International Journal of Pharmaceutical Research. 2021;13(3):1304-8. | Non-RCT |
| Alotaibi, M. K. Truncal Acne in Adolescents and Young Adults in Saudi Arabia: A Self-Reported Perception. Turkish Journal of Dermatology. 2024; 18 (4) | Non-RCT |
| Alrabiah Z, Arafah A, Rehman MU, Babelghaith SD, Syed W, Alrashidi FK, et al. Prevalence and Self-Medication for Acne among Students of Health-Related Science Colleges at King Saud University in Riyadh Region Saudi Arabia. Medicina (Kaunas, Lithuania). 2022;59(1). | Non-RCT |
| Al-Shobaili HA. Knowledge and practice of primary healthcare physicians for management of acne vulgaris in Qassim region, Saudi Arabia. The Journal of the Egyptian Public Health Association. 2013;88(1):26-31. | Non-RCT |
| Ansari, F., et al. (2022). Myths, misconceptions and attitudinal trends among patients with acne. Indian Journal of Dermatology, Venereology and Leprology: 1-6. | Non-RCT |
| Aslan Kayiran M, Karadag AS, Mutlu HH, Goldust M, Saricaoglu H. Comparison of dermatologists and family physicians in terms of prescribing antibiotics for the treatment of acne vulgaris. Dermatologic Therapy. 2020;33(6):e13973. | Non-RCT |
| Aslan Kayiran M, Karadag AS, Alyamac G, Cevirgen Cemil B, Demirseren DD, Aksoy H, et al. Social media use in patients with acne vulgaris: What do patients expect from social media? Journal of Cosmetic Dermatology. 2021;20(8):2556-64. | Non-RCT |
| Aslan Kayiran M, Karadag AS, Alyamac G, Cevirgen Cemil B, Demirseren DD, Tas Demircan Y, et al. Rates of skincare product and cosmetic procedure use in patients with acne vulgaris and the effective factors: A multicenter study with 1,755 patients. Journal of Cosmetic Dermatology. 2022;21(6):2566-76. | Non-RCT |
| Baldwin, H. and Blanco, D. and McKeever, C. and Paz, N. and Vasquez, Y. N. and Quiring, J. and Enloe, C. and De Leon, E. and Stasko, N. Results of a phase 2 efficacy and safety study with SB204, an investigational topical nitric oxide-releasing drug for the treatment of acne vulgaris. Journal of Clinical and Aesthetic Dermatology. 2016; 9 (8) | Non-RCT |
| Ballanger F, Claudel JP, Leccia MT, Auffret N, Camerati AS, Dufaux PO, et al. Truncal Acne in Adolescents and Young Adults: Self-reported Perception. Acta Dermato-Venereologica. 2023;103:adv5123. | Non-RCT |
| Batra, R. and Yogendra, M. and Gaurkar, S. and Mukhopadhyay, P. and Sen, D. and Jadhwar, S. Investigating the Use of 0.3% Adapalene/2.5% Benzoyl Peroxide Gel for the Management of Moderate-to-Severe Acne in Indian Patients: A Phase 4 Study Assessing Safety and Efficacy. Cureus. 2024; 16 (7) | Non-RCT |
| Berg M, Lindberg M. Possible gender differences in the quality of life and choice of therapy in acne. Journal of the European Academy of Dermatology and Venereology. 2011;25(8):969-72. | Non-RCT |
| Bhatia, N. and Donald, A. and Mariwalla, K. Efficacy and Consumer Perception of Topical Over-the-Counter Regimens Involving Beta-Hydroxy Acid for Acne Vulgaris. Journal of drugs in dermatology : JDD. 2025; 24 (2) | Non-RCT |
| Bhattacharya, R. and Dwibedi, N. A web-based survey to evaluate the factors associated with treatment seeking behavior in adults with acne Vulgaris. Value in Health. 2014; 17 (3) | Non-RCT |
| Callender, V. D., et al. (2014). Racial differences in clinical characteristics, perceptions and behaviors, and psychosocial impact of adult female acne. Journal of Clinical and Aesthetic Dermatology 7(7): 19-31. | Non-RCT |
| Cardona-Arias JA, Alvarez-Medina MC, Martinez-Valencia DM, Cruz-Tobon AM, Vallejo-Patino MM. Prevalence of acne in students of eighth through eleventh of el retiro-antioquia and associated perceptions. Archivos de Medicina. 2014;10(1). | Non-RCT |
| Chaudhary, R. To determine the efficacy of the doxycycline and trifarotene combination in treating acne vulgaris. International Journal of Life Sciences Biotechnology and Pharma Research. 2022; 11 (2) | Non-RCT |
| Chen CL, Kuppermann M, Caughey AB, Zane LT. A community-based study of acne-related health preferences in adolescents. Archives of Dermatology. 2008;144(8):988-94. | Non-RCT |
| Cheng CE, Irwin B, Mauriello D, Liang L, Pappert A, Kimball AB. Self-reported acne severity, treatment, and belief patterns across multiple racial and ethnic groups in adolescent students. Pediatric Dermatology. 2010;27(5):446-52. | Non-RCT |
| Chernyshov PV, Petrenko A, Kopylova V. What health-related quality of life factors influence the decision of patients with acne to visit a dermatologist? Journal of Clinical and Aesthetic Dermatology. 2018;11(7):21-5. | Non-RCT |
| Chung, E. H. and Son, J. W. and Eun, Y. S. and Yang, N. G. and Kim, J. Y. and Lee, S. and Heo, N. H. and Rhee, J. and Lee, S. Y. and Hwang, Y. and Kim, J. E. A Novel, Hand-Held, and Low-Level Light Therapy Device for the Treatment of Acne Vulgaris: A Single-Arm, Prospective Clinical Study. Dermatologic Therapy. 2023; 2023 | Non-RCT |
| Corey, K. C., et al. (2013). Self-reported help-seeking behaviors and treatment choices of adolescents regarding acne. Pediatric Dermatology 30(1): 36-41. | Non-RCT |
| de Vries F, Welbie M, Tjin E, Driessen R, van de Kerkhof P. Reallocations in acne healthcare: exploring the possible roles and added value of non-physicians by a mixed-methods study design. BMC health services research. 2021;21(1):746. | Non-RCT |
| de Vries F, Tjin E, Driessen R, Vehof H, van de Kerkhof P. Exploring patient journeys through acne healthcare: a patient perspective. Journal of Dermatological Treatment. 2022;33(4):2209-16. | Non-RCT |
| Desai KP, Martyn-Simmons C, Viner R, Segal T. Help-seeking behaviours, opportunistic treatment and psychological implications of adolescent acne: Cross-sectional studies in schools and hospital outpatient departments in the UK. BMJ Open. 2017;7(9):e016964. | Non-RCT |
| Draelos, Z. D. Application Characteristics and Patient Preference of Triple-Combination vs Layered Topicals for Acne: Split-Face Study. J Drugs Dermatol. 2024; 23 (10) | Non-RCT |
| Eichenfield, L. F. and DuBois, J. C. and Gold, M. H. and Nardo, C. J. and Draelos, Z. D. and Armas, E. and DeValle, O. and Haber, R. S. and Jones, T. and Kempers, S. E. and Knoepp, T. G. and Kuttner, B. J. and Lieberman, R. and Miller, M. L. and Stewart, D. M. and Weinberg, J. DMT310, a novel once-weekly topical treatment for patients with moderate-to-severe acne vulgaris: Results of a phase 2b randomized, double-blind, placebo-controlled trial. Journal of the American Academy of Dermatology. 2023; 89 (5) | Non-RCT |
| Eichenfield, L. F. and Gold, L. S. and Han, J. and Hebert, A. A. and Mazzetti, A. and Moro, L. and Squittieri, N. and Thiboutot, D. Integrated Short-Term and Long-Term Efficacy of Topical Clascoterone Cream 1% in Patients >=12 years of Age With Acne Vulgaris. Journal of Drugs in Dermatology. 2024; 23 (1) | Non-RCT |
| Eichenfield, L. F. and Hebert, A. A. and Gold, L. S. and Cartwright, M. and Moro, L. and Han, J. and Squittieri, N. and Mazzetti, A. Long-Term Safety and Efficacy of Twice-Daily Topical Clascoterone Cream 1% in Patients >= 12 Years of Age With Acne Vulgaris. Journal of Drugs in Dermatology. 2023; 22 (8) | Non-RCT |
| Eichenfield, L. F. and Hebert, A. A. and Harper, J. C. and Baldwin, H. and Bhatia, N. and Gold, L. S. and Kircik, L. H. and Graber, E. and Tanghetti, E. A. and Alexis, A. F. and Del Rosso, J. Q. Triple-Combination Clindamycin Phosphate 1.2%/ Adapalene 0.15%/Benzoyl Peroxide 3.1% Gel for Moderate-to-Severe Acne in Children and Adolescents. Journal of Drugs in Dermatology. 2024; 23 (12) | Non-RCT |
| Eichenfield, L. F. and Stein Gold, L. and Kircik, L. H. and Werschler, W. P. and Beer, K. and Draelos, Z. D. and Tanghetti, E. A. and Papp, K. A. and Baldwin, H. and Lain, E. and Sadick, N. and Gooderham, M. J. and Konda, A. Triple-combination clindamycin phosphate 1.2%/benzoyl peroxide 3.1%/adapalene 0.15% gel for moderate-to-severe acne in children and adolescents: Randomized phase 2 study. Pediatric Dermatology. 2023; 40 (3) | Non-RCT |
| Geetha, A. and Vishaka and Raghav, M. V. and Asha, G. S. A Comparative Study of Efficacy and Tolerability of Topical Benzoyl Peroxide versus Topical Benzoyl Peroxide with Adapalene in Mild to Moderate Acne. Indian Journal of Pharmaceutical Sciences. 2023; 85 (5) | Non-RCT |
| Gern, A. and Walter, J. and Xu, S. and Vakharia, P. P. A randomized controlled double-blinded split-face prospective clinical trial to assess the efficacy, safety, and tolerability of a novel 3-step routine compared to benzoyl peroxide for the treatment of mild to moderate acne vulgaris. Archives of Dermatological Research. 2024; 316 (6) | Non-RCT |
| Ghanem RE, Mostafa SH, Abu Hamamda NK, Khdour MR. Knowledge, attitudes, and practices of community pharmacists toward the management of acne vulgaris in Palestine: a cross-sectional study. International Journal of Dermatology. 2020;59(4):506- | Non-RCT |
| Gloor M, Eicher C, Wiebelt H, Moser G. Sociological study of acne vulgaris. II. Effect of the disease on assessment of clinical picture and motivation to treatment. H+G Zeitschrift fur Hautkrankheiten. 1978;53(24):905-13. | Non-RCT |
| Gupta A, Dhande P. Knowledge, perceptions and psychosocial impact of acne vulgaris: An Indian scenario. Journal of Clinical and Diagnostic Research. 2019;13(3):WC01-WC6. | Non-RCT |
| Hanstock TL, O'Mahony JF. Treatment seeking in young women with acne. Dermatology and Psychosomatics. 2003;4(4):194-9. | Non-RCT |
| Heidemeyer K, Bodle L, Boll S, Bossart S, Jafari SMS, Hunger RE, et al. Randomized Open-Label Trial Comparing Teledermatology vs. Face-to-Face Consultation in the Follow-Up of Patients with Mild-to-Moderate Acne. Acta Informatica Medica. 2023;31(1):31-6. | RCT |
| Jones-Caballero, M., et al. (2007). Quality of life in mild to moderate acne: Relationship to clinical severity and factors influencing change with treatment. Journal of the European Academy of Dermatology and Venereology 21(2): 219-226. | Non-RCT |
| Kaushik M, Gupta S, Mahendra A. Living with acne: Belief and perception in a sample of Indian youths. Indian Journal of Dermatology. 2017;62(5):491-7. | Non-RCT |
| Kayıran M, Karadağ A, Alyamaç G, Cemil B, Demirseren D, Demircan Y, et al. Use of complementary and alternative medicine among patients with acne vulgaris and factors perceived to trigger the disease: A multicentre cross-sectional study with 1571 patients. Indian Journal of Dermatology. 2022;67(3):311-. | Non-RCT |
| Kellett N, West F, Finlay AY. Conjoint analysis: A novel, rigorous tool for determining patient preferences for topical antibiotic treatment for acne. A randomised controlled trial. British Journal of Dermatology. 2006;154(3):524-32. | RCT |
| Khairani O, Zaiton S, Faridah MN. Do adolescents attending Bandar Mas primary care clinic consult health professionals for their common health problems? Medical Journal of Malaysia. 2005;60(2):134-9. | Non-RCT |
| Khosravi H, Zhang S, Siripong N, Moorhead A, English JC. Comparing acne follow-up: Teledermatology versus outpatient dermatology visits. Dermatology Online Journal. 2020;26(4):1. | Non-RCT |
| Kingston P, Yee D, Huang MY, Korouri E, Peterson H, Lee K, et al. Shared decision-making and satisfaction with care: a population-based study in acne patients with skin of color in the United States. Clinical and experimental dermatology. 2023. | Non-RCT |
| Kohn LL, Pickett K, Day JA, Torres-Zegarra C, Plost G, Gurnee E, et al. When is synchronous telehealth acceptable for pediatric dermatology? Pediatric Dermatology. 2022;39(2):236-42. | Non-RCT |
| Kubota Y, Shirahige Y, Nakai K, Katsuura J, Moriue T, Yoneda K. Community-based epidemiological study of psychosocial effects of acne in Japanese adolescents. Journal of Dermatology. 2010;37(7):617-22. | Non-RCT |
| Kwon HH, Yoon HS, Suh DH, Yoon JY, Park SK, Lee JH, et al. A nationwide study of acne treatment patterns in Korea: Analysis of patient preconceived notions and dermatologist suggestion for treatment. Acta Dermato-Venereologica. 2012;92(3):236-40. | Non-RCT |
| Lal DM, Mansoor M, Amin E, Memon MM, Khan MS, Talat H, et al. Knowledge attitude and practices among medical students regarding acne. Journal of the Liaquat University of Medical and Health Sciences. 2021;20(3):246-51. | Non-RCT |
| Law MPM, Chuh AAT, Lee A, Molinari N. Acne prevalence and beyond: Acne disability and its predictive factors among Chinese late adolescents in Hong Kong. Clinical and Experimental Dermatology. 2010;35(1):16-21. | Non-RCT |
| Leelavathi M, Tan H C, Puah J. W D, Apipi M, Sohami A E, Mahat N F. Acne Disability, Self Management and Help-Seeking Behaviour among Medical Students. Medicine & Health (Universiti Kebangsaan Malaysia). 2015;10(1):1-9. | Non-RCT |
| Lopez-Gehrke I, Claycomb R. Immune defence proteins (IDP) in acne vulgaris: The importance of the barrier function of skin. our experience in Mexico. Australasian Journal of Dermatology. 2015;56(4):a6. | Non-RCT |
| Luqman N, Hassan S, Asghar R, Waheed F, Khalid M, Shaheen JA, et al. Knowledge, attitude and practices about acne vulgaris among acne patients. Journal of Pakistan Association of Dermatologists. 2021;30(4):592-7. | Non-RCT |
| MacHiwala A, Kamath G, Vaidya T. Knowledge, belief, and perception among youths with acne vulgaris. Indian Journal of Dermatology. 2019;64(5):389-91. | Non-RCT |
| Margolis CF, Ramundo ML. Acne management. Primary care physician or dermatologist? Postgraduate Medicine. 1987;82(8):139-46. | Non-RCT |
| Mastrolonardo M, Bonamonte D, Karlis X, Unato FF, Mazzarella F, Romita P, et al. Acne and cosmetics: A cross-sectional, web-based questionnaire survey of the views and opinions of Italian dermatologists on the use of camouflage cosmetics in female patients. Giornale Italiano di Dermatologia e Venereologia. 2017;152(5):407-12. | Non-RCT |
| Mehta M, Kundu RV. Racial Differences in Treatment Preferences of Acne Vulgaris: A Cross-Sectional Study. Journal of drugs in dermatology : JDD. 2020;19(12):802. | Non-RCT |
| Motley RJ, Finlay AY. How much disability is caused by acne? Clinical and Experimental Dermatology. 1989;14(3):194-8. | Non-RCT |
| McEvoy, B., et al. (2003). Factors related to patient compliance in the treatment of acne vulgaris. International Journal of Dermatology 42(4): 274-280. | Non-RCT |
| Nguyen QG, Markus R, Katta R. Diet and acne: an exploratory survey study of patient beliefs. Dermatol Pract Concept. 2016;6(2):21-7. | Non-RCT |
| Nijsten T, Rombouts S, Lambert J. Acne is prevalent but use of its treatments is infrequent among adolescents from the general population. J Eur Acad Dermatol Venereol. 2007;21(2):163-8. | Non-RCT |
| Ozolins M, Eady EA, Avery A, Cunliffe WJ, O'Neill C, Simpson NB, et al. Randomised controlled multiple treatment comparison to provide a cost-effectiveness rationale for the selection of antimicrobial therapy in acne. Health technology assessment (Winchester, England). 2005;9(1):iii-212. | RCT |
| Pawin, H., et al. (2007). "Living with acne: A study of adolescents' personal experiences." Dermatology 215(4): 308-314. | Non-RCT |
| Perche P, Singh R, Feldman S. Patient Preferences for Acne Vulgaris Treatment and Barriers to Care: A Survey Study. Journal of drugs in dermatology : JDD. 2022;21(11):1191-5. | Non-RCT |
| Poli F, Auffret N, Beylot C, Chivot M, Faure M, Moyse D, et al. Acne as seen by adolescents: Results of questionnaire study in 852 French individuals. Acta Dermato-Venereologica. 2011;91(5):531-6. | Non-RCT |
| Rasmussen JE, Smith SB. Patient concepts and misconceptions about acne. Archives of Dermatology. 1983;119(7):570-2. | Non-RCT |
| Reich A, Jasiuk B, Samotij D, Tracinska A, Trybucka K, Szepietowski JC. Acne vulgaris: what teenagers think about it. Dermatology nursing / Dermatology Nurses' Association. 2007;19(1):49-64. | Non-RCT |
| Reddy, P. S., et al. (2021). Antibiotics for acne vulgaris: using instagram to seek insight into the patient perspective. Journal of Dermatological Treatment 32(2): 188-192. | Non-RCT |
| Rigopoulos D, Gregoriou S, Ifandi A, Efstathiou G, Georgala S, Chalkias J, et al. Coping with acne: Beliefs and perceptions in a sample of secondary school Greek pupils. Journal of the European Academy of Dermatology and Venereology. 2007;21(6):806-10. | Non-RCT |
| Rueda MJ. Acne Subject Preference for Pump Over Tube for Dispensing Fixed-Dose Combination Adapalene 0.1%-Benzoyl Peroxide 2.5% Gel. Dermatology and Therapy. 2014;4(1):61-70. | RCT |
| Savo I, Jorgaqi E, Vasili E, Mishtaku S, Demaj D, Jafferany M. Treatment-seeking behavior, knowledge and beliefs about acne vulgaris among adolescents: A cross-sectional study in high school students in Tirana, Albania. Dermatologic Therapy. 2020;33(4):e13500. | Non-RCT |
| Shah N, Ghatnekar S, Nambudiri VE. Variability of over-the-counter spending on acne treatment across race and demographic characteristics. Clinics in Dermatology. 2022;40(5):564-6. | Non-RCT |
| Smithard A, Glazebrook C, Williams HC. Acne prevalence, knowledge about acne and psychological morbidity in mid-adolescence: A community-based study. British Journal of Dermatology. 2001;145(2):274-9. | Non-RCT |
| Su, P., et al. (2015). Beliefs, perceptions and psychosocial impact of acne amongst Singaporean students in tertiary institutions. JDDG - Journal of the German Society of Dermatology 13(3): 227-234. | Non-RCT |
| Suh DH, Shin JW, Min SU, Lee DH, Yoon MY, Kim NI, et al. Treatment-seeking behaviors and related epidemiological features in Korean acne patients. Journal of Korean medical science. 2008;23(6):969-74. | Non-RCT |
| Suthipinittharm P, Noppakun N, Kulthanan K, Jiamton S, Rajatanavin N, Aunhachoke K, et al. Opinions and perceptions on acne:A community-based questionnaire study in Thai students. Journal of the Medical Association of Thailand. 2013;96(8):952-9. | Non-RCT |
| Tahir CM. Efficacy and adverse effects of systemic isotretinoin therapy. Journal of Pakistan Association of Dermatologists. 2011;21(1):38-42. | Non-RCT |
| Tan J, Chien AL, York JP, Layton AM. Concerns, Expectations, and Management Gaps Relating to Acne and Associated Scarring Identified from Survey Data. Journal of drugs in dermatology : JDD. 2021;20(6):600-6. | Non-RCT |
| Tan J, Beissert S, Cook-Bolden F, Chavda R, Harper J, Hebert A, et al. Evaluation of psychological well-being and social impact of atrophic acne scarring: A multinational, mixed-methods study. JAAD Int. 2022;6:43-50. | Non-RCT |
| Tan,Y. J. and Jamil, A and Gunabalasingam, P. Disease perception, treatment-seeking behaviour and psychosocial impact of acne vulgaris among university students – A cross-sectional study. Malaysian Family Physician 2025; 20 (3) | Non-RCT |
| Tanghetti EA, Kawata AK, Daniels SR, Yeomans K, Burk CT, Callender VD. Understanding the burden of adult female acne. Journal of Clinical and Aesthetic Dermatology. 2014;7(2):22-30. | Non-RCT |
| Tavecchio S, Barbareschi M, Veraldi S. What Italians think about acne: Results of a survey on 2327 acne patients and their mothers. Giornale Italiano di Dermatologia e Venereologia. 2020;155(5):642-5. | Non-RCT |
| Tuong W, Wang AS, Armstrong AW. Effect of Automated Online Counseling on Clinical Outcomes and Quality of Life Among Adolescents With Acne Vulgaris: A Randomized Clinical Trial. JAMA Dermatol. 2015;151(9):970-5. | RCT |
| Uslu G, Sendur N, Uslu M, Savk E, Karaman G, Eskin M. Acne: Prevalence, perceptions and effects on psychological health among adolescents in Aydin, Turkey. Journal of the European Academy of Dermatology and Venereology. 2008;22(4):462-9. | Non-RCT |
| Veraldi, S., et al. (2016). Role of cleansers in the management of acne: Results of an Italian survey in 786 patients. Journal of Dermatological Treatment 27(5): 439-442. | Non-RCT |
| Wisuthsarewong W, Nitiyarom R, Kanchanapenkul D, Arunkajohnask S, Limphoka P, Boonchai W. Acne beliefs, treatment-seeking behaviors, information media usage, and impact on daily living activities of Thai acne patients. Journal of Cosmetic Dermatology. 2020;19(5):1191-5. | Non-RCT |
| Yahya H. Acne vulgaris in Nigerian adolescents - Prevalence, severity, beliefs, perceptions, and practices. International Journal of Dermatology. 2009;48(5):498-505. | Non-RCT |
| Yentzer BA, Irby CE, Fleischer Jr AB, Feldman SR. Differences in acne treatment prescribing patterns of pediatricians and dermatologists: An analysis of nationally representative data. Pediatric Dermatology. 2008;25(6):635-9. | Non-RCT |
| Yentzer BA, Hick J, Reese EL, Uhas A, Feldman SR, Balkrishnan R. Acne vulgaris in the United States: a descriptive epidemiology. Cutis. 2010;86(2):94-9. | Non-RCT |
| Yeung CK, Teo LHY, Xiang LH, Chan HHL. A community-based epidemiological study of acne vulgaris in Hong Kong adolescents. Acta Dermato-Venereologica. 2002;82(2):104-7. | Non-RCT |
| Yorulmaz A, Yalcin B. Myths, Perceptions and Practices in Acne: A Study on Adolescents and Young Adults. Curr Health Sci J. 2020;46(2):111-6. | Non-RCT |
| Yousaf A, Hagen R, Delaney E, Davis S, Zinn Z. The influence of social media on acne treatment: A cross-sectional survey. Pediatric Dermatology. 2020;37(2):301-4. | Non-RCT |
